# Supplementary material for: Usual care in a multicentre randomised controlled trial of financial incentives for smoking cessation in pregnancy: qualitative findings from a mixed-methods process evaluation
Source: BMJ Open. 2022 Dec 7;12(12):e066494. doi: 10.1136/bmjopen-2022-066494 (PMC9730354; doi:10.1136/bmjopen-2022-066494)
Supplement: Supplementary data [file bmjopen-2022-066494supp002.pdf]

CPIT Qualitative Research Topic Guide
Interviews with Professionals with a Relevant Role
e.g. with pregnant smokers, cessation services
V1.0: 05/07/2017

Interviews with professionals aim to explore issues around referral and recruitment to the trial, and perceived response among participants.

Interviews will be adapted depending on respondents' experience.
The topic guide is intended to ensure coverage of key topics, whilst at the same time giving respondents the freedom to express their own feelings and views as part of an open discussion.
****

INTRODUCTION
- Provide a reminder of the purpose and main focus of the trial
- Explain that focusing on opinions and experiences (not a test)
- Provide opportunity to ask questions
- Introduce the audio-recorder, underlining the importance of confidentiality
- Ask participant to complete consent form

A. BACKGROUND: OVERVIEW OF ROLE AND RELEVANT INVOLVEMENT IN TRIAL (also warm-up questions)
- General role
- Involvement with cessation in pregnancy
- Involvement in the trial

B. CONFIGURATION OF SERVICE AND CHARACTERISTICS OF PATIENT COMMUNITY
- Nature of service in which you work: how is it structured, where is it based, what staff groupings are involved
- Links between maternity service staff and smoking cessation staff or services: including knowledge, communication, referral process, feedback
- Characteristics of patient community served: level of affluence, degree to which urban/rural; age range, ethnicity, unemployment status, other?

C. TRIAL SET UP AND SUPPORT
- Explore knowledge and experience of changes made in anticipation of trial within own and related services
  - Communication and consultation regarding trial
  - Training
  - Working with other professionals
  - Facilities including access to consultation rooms, equipment, IT
  - Perception of response of colleagues in own as well as other services
  - Other challenges or opportunities not covered
  - Any perceived shortcomings in service preparations for trial

- Explore general experience of referral and recruitment of pregnant women to the trial then focus upon elements not already discussed. *Not all will be relevant to individual experience.*

<u>Initial booking visit (Midwives)</u>
  - Asking pregnant women about their smoking
  - Taking carbon monoxide readings (CO) readings and recording

- Asking permission to send information or dicussing automatic referral (where appropriate) to NHS stop smoking services
- Explore challenges and opportunities e.g. demanding appointment session, smokers' responses, preparation for this activity

First telephone contact with NHS Smokefree Pregnancy adviser (Smoking cessation staff)

- challenges of making contact by telephone (including out of hours)
- informing about the trial
- verbal permission to forward details to the Contact Centre
- perceived clients' response to service (e.g. thoughts on attending session, challenges to
- quitting)
- perceived clients' response to trial including reasons for participation (e.g. impact of incentives, random allocation)
- Process issues e.g. capacity issues in covering appointments
- Any prior awareness of using incentives for cessation and current trial

Consent call from NHS Stop Smoking Helpline (Contact Centre staff):

- Giving information about the trial
- Obtaining verbal consent
- Introducing and discussing random allocation to intervention or control groups
- Explore views of challenges experienced or anticipated e.g. contact successes, explaining complex issues, response to random allocation, perceived effect on engaging with services.

D. SUGGESTIONS FOR IMPROVING REFERRAL & RECRUITMENT

- Engaging women in the trial
  - Terms to describe the incentive scheme to pregnant women / what are key aspects women need to know/how best communicated
  - Any thoughts on what would make it easier/more attractive for women to engage in the trial/intervention
- What have been the barriers to referral and recruitment and what have been facilitators?
- Any ideas on what could be done to address barriers and enhance or extend facilitators?
- Any additional learning?

E. OVERVIEW

- Responses to concept of incentives - respondent's views and perceptions of others' views e.g. pregnant women, professionals and general public
  - Incentives as provided in current trial [provide overview of key stages within intervention and associated incentives]
  - Perceptions of any unintended outcomes
- Thoughts on incentives becoming a standard approach for supporting pregnant smokers to quit
- Any final thoughts on trial
