# Supplementary material for: Usual care in a multicentre randomised controlled trial of financial incentives for smoking cessation in pregnancy: qualitative findings from a mixed-methods process evaluation
Source: BMJ Open. 2022 Dec 7;12(12):e066494. doi: 10.1136/bmjopen-2022-066494 (PMC9730354; doi:10.1136/bmjopen-2022-066494)
Supplement: Supplementary data [file bmjopen-2022-066494supp001.pdf]

CPIT Qualitative Research Topic Guide
Interviews with Pregnant Women V1.0 05/07/2017

One-to-one telephone interviews with pregnant women presenting to the various study centres during the trial period, including women who decide to take part in the trial and those who decline.

Aim to explore views on issues around health professionals raising smoking in pregnancy i.e. midwives, referral to SSSs, discussion of study and decisions to take part or decline. Thoughts on consent phone call from NHS Stop smoking and random allocation to intervention or control.

Interviews will be adapted depending on experience, e.g. participation or non participation; intervention or control status,

The topic guide is intended to ensure coverage of key topics, whilst at the same time giving respondents the freedom to express their own feelings and views as part of an open discussion.

****

INTRODUCTION

- Provide a reminder of the purpose and main focus of the study
- Explain that focusing on opinions and experiences (not a test)
- Provide opportunity to ask questions
- Introduce the audio-recorder, underlining the importance of confidentiality
- Ask participant to complete consent form

A. BACKGROUND: BROAD SMOKING HISTORY AND LOCAL CONTEXT (ALSO WARM-UP QUESTIONS)

- How long smoked / how many a day / how easy to stop (anticipated/experienced)
- Any previous quit attempts/relapse events (inc. any previous pregnancies)
- When baby due
- How common is smoking where you live?
    - What do people you know think about smoking?

B. INTRODUCTION TO STOP SMOKING SUPPORT AND TRIAL PARTICIPATION

Ante-natal appointment(s) with Midwife

- Talk me through your first ante-natal visit
    - How did you feel about being advised to stop smoking?
    - How did you feel about referral to Stop Smoking Services (also where appropriate, automatic or opt-out referral)
    - How did you feel about CO monitoring (breath test)
- Any discussion of smoking at subsequent ante-natal appointments

Initial contact(s) with NHS Smokefree Pregnancy Service Advisor

- Explore telephone call OR drop-in at clinic
    - Thoughts on approach of advisor to stopping smoking
    - First thoughts when heard about the study from advisor
    - Everything clear/questions answered about trial
    - Reasons why you agreed/did not agree to having your contact details passed to the trial team

- Thoughts on information sheet received about trial: enough information; any questions

Trial Enrolment

- Can you talk me through what happened after you agreed to your details being passed to the trial team?
  - Thoughts and feelings about consent phonecall
- Why did you agree/decline to take part in the trial?
  - Were there any aspects of the trial that meant you were more or less likely to take part?
- Understanding of selection to intervention or control group
- Feelings about selection to intervention or control group
- Overall feelings about decision to take part after consent call

C. SUGGESTIONS FOR IMPROVING SERVICE AND/OR THE TRIAL

- Elements of the stop smoking service that are most/least useful – why?
- Things that might help more / improve the stop smoking service
- Promoting the incentive scheme
  - Key messages / ideas on ‘selling points’ for women
- Any thoughts on what would make it easier/more attractive for women to take part in the trial
- Anything else you would change or improve?

D. OVERVIEW

- Impact of being part of a trial, e.g. on motivation, impact of being a ‘control’
- Any other/final thoughts?
