# Supplementary material for: Usual care in a multicentre randomised controlled trial of financial incentives for smoking cessation in pregnancy: qualitative findings from a mixed-methods process evaluation
Source: BMJ Open. 2022 Dec 7;12(12):e066494. doi: 10.1136/bmjopen-2022-066494 (PMC9730354; doi:10.1136/bmjopen-2022-066494)
Supplement: Supplementary data [file bmjopen-2022-066494supp003.pdf]

CPIT 3 Process Evaluation

Nodes\\Revised Coding Framework

Coding Framework revised after testing by JM and IU August 2019

Name	Description
01, Job Role, professional background and experience	
a) Description of job role and relevant training	Including length of time in current post and professional background
b) Role within the trial	Including feelings about role within the trial
e) Professional standards or codes of conduct	
f) Importance of research evidence to role or practice	
02, Trial introduction and set-up	
a) How and when first heard about trial and from whom	
b) Perspectives of the trial team	
c) Initial thoughts and feelings about using incentives to encourage smoking	Including interest in the trial

McKell J, *et al. BMJ Open* 2022; 12:e066494. doi: 10.1136/bmjopen-2022-066494

Name	Description
cessation	
d) Initial thoughts and feelings about service or employer being involved with trial	
e) Decision-making about involvement in the trial	
f) Expectations of the trial	Including thoughts on execution of the research - also thoughts on CPIT phase 2 results
g) Preparations and training for CPIT	
03, Routine Service Characteristics	
a) Nature of booking appointment and midwifery care generally	Including impact of national policies on midwifery care.
b) Discussion of smoking within booking appointments	Where relevant to discussion of smoking also mention of CO monitoring
c) CO monitoring	
d) Criteria for and method of referral plus how women are first contacted by SSS	Also includes the typical number of referrals received each month by the service
e) Approach to and nature of support	Including level of engagement from women (prior to the trial). Also includes approach of midwives to

McKell J, *et al. BMJ Open* 2022; 12:e066494. doi: 10.1136/bmjopen-2022-066494

Name	Description
from smoking cessation service	support e.g specialist substance use midwives
f) Access to NRT and ability to prescribe directly to clients	
g) Monitoring of referral, engagement and outcomes	
h) Links between maternity staff and SSS	Including perspectives of the other group
i) Description and history of service or employing organisation	Also, description of management structures and organisational change prior to the trial. Description of how they feel about service including possible ownership. Also relationship with women - level of engagement
j) Discussion of local or national strategies with relevance to service or individual role	
04, Delivery and process of trial (feasibility)	
a) Introducing the trial to women referred	Including checking for eligibility
b) Consent call from ECHO	Including thoughts on randomisation to one of two groups
c) Face to face contact with adviser and setting a quit date	

Name	Description
d) Smoking status phone calls or research nurse visits for CO monitoring and triggering of incentives	
e) End of pregnancy smoking status, CO monitoring and cotinine testing	
f) Final smoking status check at 6 months post partum	
g) Client receiving or using financial incentives at key stages, practical issues	
h) Timing issues and any impact of delays	
05, Impact of the trial on services and women	
a) Maternity Service	
b) Smoking Cessation Service	
c) Women	
06, Making the trial work	
a) Information sharing and communication between trial staff and	

Name	Description
SSS	
b) Cooperation and compromise	
c) Support from the trial to the SSS	
07, Incentives and motivation	
a) Perspectives of acceptability of using incentives to promote smoking cessation in pregnancy	
i) Any awareness of incentives being used to change other behaviours	
ii) Feelings regarding the use of incentives	
iii) Type of incentive i.e financial versus other	
iv) Amounts and staged approach	
v) Thoughts on possible impact including length of time offered	
b) Perceived impact on client motivation to engage, to quit, to stay	

McKell J, *et al. BMJ Open* 2022; 12:e066494. doi: 10.1136/bmjopen-2022-066494

Name	Description
quit	
i) Overview of motivating factors for quit	
ii) Perceived impact of incentives	Including extent to which there has been greater engagement from women or the types of women who are accessing support
iii) Relative importance of incentives in context of other intervention factors	e.g. Role of smoking cessation adviser or CO monitoring
iv) Relative importance of incentives in context of other personal factors	e.g health, saving money, stress, smoking by partner or family and friends
v) Women's feedback on incentives	Including what incentives have been used on
vi) Any impact on professional appreciation of using incentives	i.e as a selling point
08, Local context and characteristics of population served by smoking cessation service	
a) Geography in terms of rural or urban setting	

Name	Description
b) Socio-economic background	
c) Age	
d) Ethnic mix	
f) Other important characteristics of local population	
09, Recommendations for improving the trial and learning for future rollout	
a) Recommendations to improve recruitment to the trial	
b) Recommendations to change type, amount or timing of incentives	
c) Recommendations to improve follow up of participants including monitoring	
d) Learning useful for future rollout from perspective of participants	
e) Learning useful for future rollout from perspective of smoking cessation service	

Name	Description
f) Views or suggestions for the process evaluation	
g) Learning points for running a trial within a service or organisation	
h) What worked in Glasgow and comparison with current sites	
10, Impression of Interview	Manner and tone of participant
11 Miscellaneous	
12 Barriers to Quitting	
13, Experience of supporting pregnant women to stop smoking*	
14, Progress of trial*	
14.1 Changes to the trial process	
15, Working with (Name of call centre company managing trial consent process)*	

* New nodes added by JM after revised framework was finalised in August 2019

McKell J, *et al. BMJ Open* 2022; 12:e066494. doi: 10.1136/bmjopen-2022-066494
