# Supplementary material for: Usual care in a multicentre randomised controlled trial of financial incentives for smoking cessation in pregnancy: qualitative findings from a mixed-methods process evaluation
Source: BMJ Open. 2022 Dec 7;12(12):e066494. doi: 10.1136/bmjopen-2022-066494 (PMC9730354; doi:10.1136/bmjopen-2022-066494)
Supplement: Author's manuscript [file bmjopen-2022-066494.draft_revisions.pdf]

BMJ Open is committed to open peer review. As part of this commitment we make the peer review history of every article we publish publicly available.

When an article is published we post the peer reviewers' comments and the authors' responses online. We also post the versions of the paper that were used during peer review. These are the versions that the peer review comments apply to.

The versions of the paper that follow are the versions that were submitted during the peer review process. They are not the versions of record or the final published versions. They should not be cited or distributed as the published version of this manuscript.

BMJ Open is an open access journal and the full, final, typeset and author-corrected version of record of the manuscript is available on our site with no access controls, subscription charges or pay-per-view fees (http://bmjopen.bmj.com).

If you have any questions on BMJ Open's open peer review process please email info.bmjopen@bmj.com

BMJ Open

Usual care in a multi-centre randomised controlled trial of financial incentives for smoking cessation in pregnancy: qualitative findings from a mixed methods process evaluation

Journal:	*BMJ Open*
Manuscript ID	bmjopen-2022-066494
Article Type:	Original research
Date Submitted by the Author:	10-Jul-2022

Complete List of Authors:	McKell, Jennifer; University of Stirling<br>Harris, Fiona M.; University of the West of Scotland<br>Sinclair, Lesley; University of York, Department of Health Sciences<br>Bauld, Linda; University of Edinburgh, Usher Institute of Population Health Sciences and Informatics<br>Tappin, David; University of Glasgow, Child Health<br>Hoddinott, Pat; University of Stirling, Nursing, Midwifery and Allied Health Professional Research Unit

Keywords:	QUALITATIVE RESEARCH, PUBLIC HEALTH, Maternal medicine < OBSTETRICS

SCHOLARONE™
Manuscripts

I, the Submitting Author has the right to grant and does grant on behalf of all authors of the Work (as defined in the below author licence), an exclusive licence and/or a non-exclusive licence for contributions from authors who are: i) UK Crown employees; ii) where BMJ has agreed a CC-BY licence shall apply, and/or iii) in accordance with the terms applicable for US Federal Government officers or employees acting as part of their official duties; on a worldwide, perpetual, irrevocable, royalty-free basis to BMJ Publishing Group Ltd ("BMJ") its licensees and where the relevant Journal is co-owned by BMJ to the co-owners of the Journal, to publish the Work in this journal and any other BMJ products and to exploit all rights, as set out in our licence.

The Submitting Author accepts and understands that any supply made under these terms is made by BMJ to the Submitting Author unless you are acting as an employee on behalf of your employer or a postgraduate student of an affiliated institution which is paying any applicable article publishing charge ("APC") for Open Access articles. Where the Submitting Author wishes to make the Work available on an Open Access basis (and intends to pay the relevant APC), the terms of reuse of such Open Access shall be governed by a Creative Commons licence – details of these licences and which Creative Commons licence will apply to this Work are set out in our licence referred to above.

Other than as permitted in any relevant BMJ Author's Self Archiving Policies, I confirm this Work has not been accepted for publication elsewhere, is not being considered for publication elsewhere and does not duplicate material already published. I confirm all authors consent to publication of this Work and authorise the granting of this licence.

For peer review only

Usual care in a multi-centre randomised controlled trial of financial incentives for smoking cessation in pregnancy: qualitative findings from a mixed methods process evaluation

Jennifer McKell[1], Fiona M. Harris[2], Lesley Sinclair[3], Linda Bauld[4], David Tappin[5], Pat Hoddinott[1]

[1]University of Stirling, Stirling, UK

[2]University of the West of Scotland, Paisley, UK

[3]University of York, York, UK

[4]University of Edinburgh, Edinburgh, UK

[5]University of Glasgow, Glasgow, UK

Corresponding author: Jennifer McKell, Institute for Social Marketing and Health, University of Stirling, Stirling, FK9 4LA (j.e.mckell@stir.ac.uk)

Word Count = 5043

For peer review only

For peer review only - http://bmjopen.bmj.com/site/about/guidelines.xhtml

ABSTRACT (WORD COUNT:291)

Objectives

Financial incentives are recommended to aid smoking cessation in pregnancy, but contextual variations in smoking cessation support could affect effectiveness. A prospective process evaluation of the Cessation in Pregnancy Incentives Trial (CPIT III) examined barriers and facilitators to cessation support (usual care) for pregnant women who smoke, prior to knowing outcomes at trial sites.

Design

Longitudinal case studies of five of seven CPIT III trial sites informed by realist evaluation.

Setting

A stop smoking service (SSS) serving a maternity hospital constituted each case study, located in three UK countries.

Participants

Data collection included semi-structured interviews with trial participants (n=22), maternity (n=13) and SSS professionals (n=18); and site observations (n=48).

Results

Cessation support (usual care) for pregnant women varied in amount, location, staff capacity, flexibility and content across sites and highlighted multiple barriers and facilitators. SSS staff capacity was important to avoid gaps in support. Co-location and good working relationships between maternity and smoking cessation professionals enabled prioritisation and reinforced the importance of smoking cessation. Sites with limited use of carbon monoxide monitoring reduced opportunities to identify smokers while inconsistency around automatic referral processes prevented the offer of cessation support. SSS staff co-located within antenatal clinics were available to women they could not otherwise reach. Flexibility around location, timing and tailoring of approaches for support, facilitated initial and sustained engagement, and reduced the burden for women.

Conclusions

Trial sites faced varied barriers and facilitators to delivering cessation support, reflecting the heterogeneity in usual care. If financial incentives are more effective with concurrent smoking cessation support, sites with fewer barriers and more facilitators would be expected to have more promising trial outcomes. Future reporting of trial outcomes will assist understanding how generalisable incentives are across a wide range of usual care settings.

For peer review only

STRENGTHS AND LIMITATIONS

- This study uniquely examines, in depth, usual care provided during a multi-centre trial of financial incentives for smoking cessation in pregnancy
- Use of site observations of usual care delivery, environment and interviews with trial participants and staff allowed exploration of multiple perspectives on usual care and the trial.
- Anonymous presentation of case study data allows for a more candid discussion of barriers and facilitators within sites.
- Case study sites represented populations with a majority white British background. Barriers and facilitators to cessation support in other more diverse populations may differ.
- Barriers and facilitators relating to communication skills e.g., the motivational techniques used by SSS staff to support cessation in pregnant women were not considered.

For peer review only

INTRODUCTION

Smoking in pregnancy raises the risk of serious consequences for mothers and babies, including miscarriage, still birth, prematurity, birth defects, and infant death[1]. Women who continue to smoke can lose on average 10 years of life whereas those who give up permanently by age 40 during their childbearing years are likely to have a normal lifespan[2]. Identifying effective and cost-effective ways to help more women to quit smoking during pregnancy remains vital, and evidence supports the use of financial incentives[3]. In 2021, the UK's National Institute for Health and Clinical Excellence (NICE) recommended the use of financial incentives to help pregnant women to quit smoking[4].

Alongside effectiveness and cost effectiveness for behavioural interventions it is important to understand how complex interventions work[5]. Support received by pregnant women alongside incentives may be a factor influencing effectiveness[6]. A pilot study in Scotland found that participants most successful in their quit attempts were those who perceived financial incentives as 'part of a wider reward structure' involving regular engagement with specialist support[7]. Similarly, Mantzari et al (2012) found that cessation support held greater importance for incentivised compared to non-incentivised women, with the former particularly motivated by 'being monitored'[8]. This evidence followed the publication of NICE guidelines in 2010, recommending 'intensive and ongoing support' of pregnant women by NHS smoking cessation services as part of usual care[9].

Offering smoking cessation support alongside financial incentives was a key characteristic of the single centre Cessation in Pregnancy Incentives Trial (CPIT II)[10]. Participants at the end of pregnancy receiving the addition of incentives were two and a half times more likely to be abstinent compared with SSS support alone (22.5%, 69/306; versus 8.6%, 26/303). The CPIT III trial commenced in 2018, replicating the CPIT II intervention and trial design in seven sites across 3 UK countries. CPIT III aimed to establish, the effectiveness, cost effectiveness and generalisability of adding the offer of financial incentives to usual care to stop smoking during pregnancy[11].

The trial protocol[11] included a mixed method process evaluation to identify and understand differences in usual care for pregnant smokers; the geographic and population mix of trial sites and their relationship to trial conduct and outcomes. The process evaluation had two broad aims:

1. To reduce the risk of the trial failing due to poor trial recruitment, by understanding contexts of identification and referral to SSS in each area, and

For peer review only - http://bmjopen.bmj.com/site/about/guidelines.xhtml

2. To understand the varied contexts in which SSSs operate so that barriers and facilitators could be identified.

This paper addresses the second aim by setting out the barriers and facilitators to providing SSS (usual care) at 5 of 7 trial sites. An a-priori proposition underpinned the process evaluation:

> Sites would vary in the usual care provided for pregnant women who smoke and therefore trial outcomes would vary by site, indicating that maternity and SSS interact with the offer of financial incentives.

METHODS

Process Evaluation Design

A longitudinal, mixed methods case study design, informed by realist evaluation, formed the basis of the embedded process evaluation[5 12 13]. The case studies, defined as a trial site (an SSS serving a maternity hospital), included 5 of the 7 trial sites representing diverse service configurations. Being one of the first five sites to open to recruitment was the criteria for selection of case study sites, though the fifth site chosen opened later than expected. All case study sites were selected independent of the main trial team. Methods and findings described in this paper relate to qualitative examination of usual care. Further details on the design of the CPIT III process evaluation are reported elsewhere[11].

Patient and Public Involvement

Two CPIT II participants and members of the UK Centre for Tobacco and Alcohol Studies (UKCTAS) smokers' panel were involved in planning CPIT III; and a patient representative was included in the CPIT III trial steering committee.

Data Collection

Case study data collection occurred in 2 stages. Stage 1 (October 2017 to December 2019) established the context for the trial locally via observation of usual care of pregnant smokers and informal interviews with maternity and SSS staff. Stage 2 (July 2018 to September 2020) involved qualitative interviews with trial participants, maternity and SSS professionals to explore experiences and perceptions relating to the trial and usual care. Concurrent to stages 1 and 2, the process evaluation team recorded trial staff perspectives relating to case study sites from trial team meetings and informal discussions with site-based trial staff. Case study specific data sources are detailed in Table 1. To protect the anonymity of professionals and participants case study sites are referred to as A-E. All trial site data sources are detailed in Table 2.

For peer review only - http://bmjopen.bmj.com/site/about/guidelines.xhtml

Table 1: Case study specific data sources (n=5)

Data Source	Site A	Site B	Site C	Site D	Site E	Total
Observations of maternity booking or other early ante-natal appointments	2	2	2	5	3	14
Observations of smoking cessation consultations	3	3	2	2	2	12
Interviews with maternity professionals	2	5	3	3	0^	13
Interviews with SSS professionals	4	3	3	5	3	18
Interviews with trial participants	2	4*	6	5	5	22
Informal discussions with local trial team members (number of trial team members consulted)	3(1)	2(1)	5+(3)	5 (2)	1(1)	16
Total	16	19	21	25	14	95

*Includes 2 interviewees who indicated an interest in the study but were not randomised;

^It was not possible to conduct interviews with maternity staff due to the onset of the Covid-19 pandemic and ongoing pressures up to the end of data collection in December 2020;

+Includes one semi-structured formal interview with local research lead. Two other trial team members consulted 4 times.

Table 2: Data sources for all trial sites (n=7)

Data Source	N
Informal discussions with Trial manager	6

For peer review only - http://bmjopen.bmj.com/site/about/guidelines.xhtml

Trial Management Working Group (TMWG) meetings	31

Data collection was undertaken by JM with assistance from IU (please see acknowledgements) both female qualitative researchers with experience of research into smoking cessation and pregnant women. Telephone interviews with trial participants ranged from 9 to 36 minutes duration. Sample sizes at sites were proportionate to the numbers recruited and aimed to achieve maximum diversity in participant age, ethnicity and socio-economic characteristics in sites with higher numbers. Two women who consented to contact about the trial but did not consent to be randomised were interviewed but elicited little additional data. Interviews with professionals, were conducted either face to face or via telephone and lasted on average 30 minutes.

Analysis

Analysis of interview transcripts and field notes was supported by QSR Nvivo (v12) software and drew on the Framework method[14 15] informed by realist evaluation. An initial coding framework was created, tested and developed by JM and IU to produce a finalised version (supplementary file). Familiarisation with data, indexing and coding led to the interpretive phase where Context Mechanism Outcome (CMO) configurations were identified. CMOs were developed with input from the wider process evaluation team (PH and FH), which included both social science and clinical input. The 'a priori' proposition was then revisited in the light of our findings, to inform the discussion.

Participant confidentiality was important to facilitate honest and information rich accounts of usual care. Staff job titles within SSS varied greatly across the sites, often reflecting the occupation of staff. In some sites, those providing cessation support were specialist midwives, while in another, nurses delivered cessation support. In two sites, support was delivered by 'facilitators' or 'wellbeing workers', with no clinical background. To minimise jigsaw identification of people or sites, quotes from professionals that deliver cessation support are referred to using the generic term 'SSS professional', regardless of occupational background, and 'senior' used for individuals with line management responsibility. Any potentially identifying information has been removed.

For peer review only

RESULTS

Usual care settings varied considerably (Box 1). Barriers and facilitators to delivering SSS support during a financial incentives trial are then presented, with CMO configurations to highlight the implications for usual care.

Box 1: Usual care settings in CPIT III trial sites

- **Site A**: Smoking cessation support was provided by a specialist midwifery led service that also offered weight management. The service was located and managed within a hospital maternity unit serving a large geographical area. Local government funding meant that only women living within relevant boundaries were eligible. Most consultations took place within the maternity unit, but others occurred at a general practitioners' (GP) surgery at a distant location, to enable local consultations. Geographic areas included low and high levels of deprivation with both urban and rural locations.

- **Site B:** A specialist midwifery service provided SSS, located in a maternity hospital with both consultant and midwifery care. The hospital was in a town that also served surrounding areas. The service was initiated with funding from a statutory public health body but subsequently transferred to the local NHS. Both high and very low levels of deprivation were present.

- **Site C:** Smoking cessation support was provided by a specialist midwifery service located in two hospitals within a city, featuring mixed levels of deprivation. One hospital offered consultant led maternity care and access to various clinical specialities while the other hospital offered midwife led care in a specially designed unit. In each hospital, the SSS occupied a dedicated consulting room, one located in an ante-natal clinic and the other close by. The service was funded by a statutory public health body with line management provided by NHS health improvement professionals.

- **Site D:** A specialist nurse service provided cessation support to the general population living in both urban and rural environments, with mixed deprivation. Support was provided in a range of settings, including pre-arranged or drop-in appointments at community venues during the day and evening. Referrals were also received regarding hospital in-patients who could be seen on the ward or at home, following discharge. Home visits were offered to people who had difficulty attending community venues. SSS management and funding was held by NHS health improvement.

- **Site E**: SSS was situated outside the NHS in a local government organisation and funded by a public health budget. Local council staff provided advice and support for health-related behaviour change to a general population. Alongside support for smoking cessation, the service also supported individuals in relation to alcohol use, weight management and physical activity.

Barriers and facilitators to providing usual care in CPIT III trial sites

Case studies highlighted barriers and facilitators to providing usual care particularly professional relationships, and service capacity. Barriers and facilitators were also identified in maternity services' ability to identify smoking in pregnancy and SSS ability to engage women in cessation

For peer review only - http://bmjopen.bmj.com/site/about/guidelines.xhtml

support, as well as features of that support, as described below. Each section concludes with the CMO configuration.

SSS capacity

Limitations on staff capacity (number, workload, absence cover, unfilled vacancies) within trial sites affected cessation support. In site C, capacity issues arose during periods of long term leave among key staff who were not quickly replaced or on the same number of hours. An intervention participant noted the impact of this staff shortage saying she would have liked more support from the study team.

> *I wasn't getting enough phone calls I don't think, […], obviously because I wasn't getting the [SSS professional] support I would have liked you know a wee phone call just, not like a formal phone call to say oh you are going to get a voucher, but just like a wee phone call to say "oh how are you doing?;[…]", I know that's not like your job or whatever but I think that would have made it a wee bit more easier (Intervention participant, Site C)*

The SSS in site D experienced numerous staff leaving and slow recruitment to posts but these difficulties were not reflected in participant or SSS professionals' perspectives. Yet evidence suggested that the service may be struggling with capacity. Trial staff noticed increasing numbers of participants being referred to local pharmacies for support, plus inconsistent CO monitoring, per protocol, in intervention participants to verify smoking abstinence 4 weeks after setting a quit date.

Staff capacity issues were less prominent in other sites but a SSS manager in site E (supporting multiple behaviour change) noted that staff numbers had reduced by more than half despite increasing demand:

> *[name of SSS] […] at the time, I think, had 41.5 full-time equivalent employees. So, we're talking about a vast big service. I think it was like £1.1 million pounds the service cost and now I think we've got 16.5 full-time equivalents, (…). So, we've slowly got smaller and smaller, and I would say that the demand on our service is far, far, higher than it ever was when we were 41.5 equivalent employees. (Senior SSS professional, Site E)*

Problems with capacity in SSS (C) reduced the time and attention staff could provide (M) increasing the likelihood of sub-optimal cessation support for pregnant women (O)

Communication and connections between midwives and SSS professionals

Shared working environments and positive, constructive relations between maternity and SSS were beneficial. Midwives and SSS professionals in three sites were co-located in the same hospital, often within easy reach, and such proximity allowed development of close, amiable working relationships. In site A, the SSS was embedded within the maternity unit, as demonstrated by efforts to extend CO monitoring in midwife appointments:

> *…when we brought in carbon monoxide and every opportunity we see them … everyone [midwives] initially thinks: 'oh my God that is extra work' and we go: 'well you are already doing their blood pressure and dipping their urine so by the time you've done that, you know, it won't take you that much longer', and everyone has just got used to it… they sort of, are used to us saying oh we are doing this now and explaining it… (SSS professional, Site B)*

Proximity working, however, was no guarantee of good working relationships. Midwives and SSS staff in one site shared consultation rooms and corridors, but gaps existed between them in relation

For peer review only - http://bmjopen.bmj.com/site/about/guidelines.xhtml

to approaches to smoking cessation, joint working, and communication. A senior SSS staff member highlighted mismatched priorities between maternity and SSS staff.

> *Community midwives what they say…yes there are some that are still perhaps not presenting it in the way that would benefit (…) the women are saying 'oh no I don't want to quit. I don't want to see them' and rather than perhaps being more positive about the [SSS] service and saying they'll have a good chat with you about the benefits of quitting or whatever. They are just informing us that these women don't want to be approached. (Senior SSS professional, Site A)*

In two sites, maternity and SSS staff never or only occasionally shared the same working environment, despite both working within community settings. The extent of links between staff groupings was sufficient for most but one SSS professional said they would like midwives' support to engage pregnant smokers.

> *if we could go and see them [midwives] and say look that girl hasn't turned up and she's coming back, if we can't get her on the phone, it might be helpful if they speak to them then. (…) So that they're getting the advice from, at each contact basically (…)* (SSS professional, Site D)

Co-location and good working relationships between maternity and smoking cessation professionals (C) prioritises and reinforces smoking cessation (M) optimising the importance of quitting for women (O).

Identifying pregnant smokers

Identification of smokers took place during initial appointments with midwives. Women were asked if they smoke and provided a carbon monoxide (CO) breath test. This was requested of all pregnant women, and not only those who self-reported smoking, to detect CO exposure and provided further opportunity to identify smokers. All sites operated an opt-out referral policy which meant that pregnant women who self-reported as smokers/recent ex-smokers or exceeded a particular CO level were automatically referred to SSS.

The role and level of implementation of CO monitoring varied by site. There was clear expectation at some sites that midwives would undertake CO monitoring at every ante-natal appointment, whereas in others, CO monitoring only occurred at initial appointments. In most sites, exceeding a particular CO level, usually of 4 parts per million or above, triggered referral to SSS for both self-reported non-smokers and smokers. In contrast, at one site, self-reported non-smokers with high CO levels were not referred but recommended for future monitoring. This suggests the following CMO configuration:

Limitations on the role and use of CO monitoring within sites (C) reduced the opportunities for midwives to identify smoking in pregnancy (M) and in turn reduced opportunities to offer cessation support (O).

Differences in interpretation of automatic referral were evident among midwives suggesting inconsistency in opportunities for smoking cessation support. Discussion of automatic referral with staff across and within sites provided contrasting descriptions of opt-out referral taking place unless a woman particularly objected versus automatic referral based on specified criteria alone:

For peer review only - http://bmjopen.bmj.com/site/about/guidelines.xhtml

> *I haven't referred anybody against their will kind of thing, you know? We do try to treat it as more of a kind of opt out service so (…) So it's more they would need to kind of say, you know what I mean, instead of us just offering would you like this or not (Midwife, Site D)*

> *It's a mandatory referral at booking, (…) I always say you will get a phone call and it's their choice then as to whether they do, because we refer everybody. Sometimes a woman will say 'I am definitely not going to be engaging with them'. And then on the referral we will put (…) 'does not want to engage', so that's the end of it really but at least the team know about them (Midwife, Site B)*

This suggests: Authentic automatic referral (C), ensures that all women are approached and informed about availability of SSS (M) and raises the possibility of SSS engagement (O).

Engagement in cessation support

SSS used multiple methods to engage pregnant smokers following maternity referral, including phone-call, text and letter but cessation staff acknowledged that often women could not be reached or were lost to follow up. Access to ante-natal clinics, however, gave cessation staff an advantage in contacting women. Staff who worked within, or close to, ante-natal clinics benefitted from opportunities to approach women in person.

> *…we are fortunate here in that we see the majority of women coming through the clinic either here or at one of the outlying clinics and for their initial scan. And so, if they're not picking the phone up to us then…we will more than likely see them when they attend for their scan and be able to have a discussion with them about smoking in pregnancy and whether they are ready to quit. (Senior SSS professional, Site A)*

Sites that enabled cessation advisers to approach women face to face at ante-natal clinics (C) provided a sense of immediacy for smoking cessation support and reduced the potential for avoidance (M), increasing the opportunities to offer support (O)

SSS ability to tailor the offer of support to optimise appeal to pregnant women also appeared important for engagement. In two sites, SSS saw opportunities in the way that smoking in pregnancy is discussed. Here staff advocated for initial conversations to emphasise exposure to CO rather than stopping smoking. A SSS professional in site C described a tailored approach for reluctant referrals.

> *it's just getting the wording right, (…) sometimes I would talk about you know, "you had your booking appointment the other day, how did you get on?, Was your scan okay?, great, I see one of your tests, your CO level was very high, it was into the red there, we'd be a bit worried there for your pregnancy, you are a smoker is that right?" (…) it's not like: [SSS professional]: "I am here to help you stop smoking"; [hypothetical woman]: "well, I don't want to stop smoking", hang up the phone." (SSS professional, Site C)*

At site D, SSS were unable to engage a trial intervention group participant who declined support due to her perception of available support. The woman felt self-conscious of being a young pregnant smoker attending a pharmacy or a 'group': '*some people are too embarrassed to go to the groups*'; though the latter was an old SSS term for a drop-in session.

SSS' ability to customise its offer of cessation support (C) to maximise appeal to individuals with different perspectives and preferences (M) facilitated initial engagement with cessation support (O).

For peer review only - http://bmjopen.bmj.com/site/about/guidelines.xhtml

Features of Cessation Support

SSS' ability to offer flexibility regarding consultations at suitable times and convenient locations made it easier for women to engage. Participants spoke of the value of SSS provided near to where they lived or via home visits.

> *I can phone her [SSS professional] if I'm stuck at any point. But the good thing is as well is if there's a time that I'm unwell she can come out to me, I just phone her. (Intervention Group Participant, Site D)*

However, SSS were not always able to provide convenient access. A SSS located in a maternity unit did not have a dedicated consulting room and could only offer afternoon appointments after ante-natal clinics. Travelling into the maternity unit was also a challenge for women living in more remote areas.

> *…certainly with the follow-ups because those appointments aren't quite so long, so women are quite reluctant to get on a bus for 45 minutes to have…to see one of us for 20 minutes. (Senior SSS professional, Site A)*

Site E SSS was able to offer weekly drop-in sessions in a community centre, one of several service venues, but consultations were limited to 15 minutes. The service manager noted less flexibility over the location and timing of appointments if a longer one-to-one consultation was required.

Providing cessation support in accessible locations and/or at convenient times (C) facilitated engagement and minimised the commitment required from women (M) increasing opportunities for sustained engagement in support (O)

How nicotine replacement therapy (NRT) was provided also had an impact on outcomes. A lack of directly provided NRT in some SSS caused delays to quit attempts. While some SSS were able to provide NRT directly, others had to refer women to their GP or linked pharmacies for NRT.

> *So, we direct the email, the prescription request, to the doctor's surgery to guarantee it gets there. (…) We used to give them [women] it and they'd be like oh we didn't' make it to the chemist and they've still got it a week later and stuff like that. […] They can pick that up within forty-eight hours, but we do put urgent, so we can phone the doctors and ask if it can be quicker, if they want to try to get that quit date set quicker. (SSS professional, Site A)*

Provision of NRT at smoking cessation consultations (C) ensures immediacy access to pharmacological support (M) enhancing pregnant smokers' ability to address nicotine withdrawal (O).

Some SSS showed a willingness to adapt to women's needs and preferences through innovation around support. Examples included extra support when dealing with low mood and depression; suggesting the use of e-cigarettes and introducing cessation support for significant others.

> *…we realised that one thing that we were coming up against quite a lot was problems with mental health and general wellbeing. So, we did an audit of our patients that were being referred into us and 50% to 60% of women that were being referred into our service had either mental health issues or general wellbeing issues […] we also run a class with [charity] […] So it all talks about mental health and antenatal depression, postnatal depression, things to look out for… (SSS professional, Site C)*

For peer review only - http://bmjopen.bmj.com/site/about/guidelines.xhtml

SSS willingness to innovate (C) extended the variety of support options available to women (M) increasing opportunities to identify effective cessation support (O).

DISCUSSION

Main findings

Cessation support provided as usual care by maternity services and SSS across trial sites varied substantially in terms of staff capacity and relationships, how services identified pregnant women who smoke, and their ability to engage, support and help women to quit.

Limitations on staff capacity in particular sites affected the amount and type of cessation support available to women. Close contact and good relationships between midwives and SSS were generally beneficial, but in some sites, there was only limited contact and cohesiveness.

CO monitoring of pregnant women throughout pregnancy was standard procedure in some sites but not others and this limited identification. Automatic opt-out referral of women who were smoking or exceeded a CO threshold was official policy across all sites but differences in interpretation meant that some women who smoked were not referred. Working within or close to ante-natal clinics enabled SSS to approach women they may otherwise struggle to reach. SSS found benefit in tailoring their approach to take account of women's perspectives and preferences. Women valued cessation support consultations held in accessible locations at a convenient time of day, but some SSS were unable to offer this flexibility. Delays to women commencing quit attempts were identified when SSS could not provide NRT directly. Willingness and capacity by some sites to offer innovative approaches provided women with a greater variety of options and facilitated engagement with support.

Comparison of findings with other studies

This process evaluation is unique in detailing site variation in usual care within a randomised controlled trial of financial incentives for smoking cessation in pregnancy. Usual care was described as variable in a previous cluster randomised controlled trial of a self-help smoking cessation intervention for pregnant women, however no detail was provided, and the intervention was found to be ineffective[16]. An implementation study which examined the introduction of BabyClear, an intervention involving universal CO monitoring in maternity booking appointments and an opt-out referral pathway, found that smoking cessation referrals and the proportion of women who had quit smoking by the time of birth in implementation sites increased[17]. A process evaluation identified barriers and facilitators to implementation that echoed findings in our study including the benefits of partnership working between midwives and SSS, flexible appointments and inconsistency of CO monitoring[18 19]. In contrast to CPIT III, the authors identified midwives' reservations around discussing smoking with pregnant women. A study of the development and implementation of an intervention to help young pregnant women to quit smoking found that home visits, sensitivity to inequalities, and direct provision of NRT were important facilitators to cessation[20]. The authors also considered support techniques provided by SSS and found motivational interviewing to be beneficial.

Limitations

The study had a number of limitations. These included the predominance of the white British populations served by the trial sites, meaning that barriers and facilitators are likely to differ for population groups not included in the study. There may have been issues with delivering behavioural support when English is not the first language, or with differing cultural attitudes and behaviours

For peer review only - http://bmjopen.bmj.com/site/about/guidelines.xhtml

related to smoking in pregnancy if a more diverse participant group had been included. In addition, resources did not permit more detailed data collection, observations and analysis of communication techniques regarding SSS communication with women.

Further work

CPIT III compared smoking cessation rates towards the end of pregnancy in women receiving usual care plus financial incentives versus usual care alone. However, with usual care among sites varying substantially, it raises the question 'what influence does this variation have on the effectiveness of financial incentives?'. Based on prior evidence that suggest cessation support could be a confounder for incentives[6], the findings of this process evaluation, help to understand the generalisability of CPIT III findings. This paper was completed prior to the research team analysing or knowing quantitative trial outcomes by site. This prospective exploratory process evaluation therefore minimises any bias that could arise from retrospective analysis once trial outcomes are known. The finding that CPIT III trial sites had differing characteristics and balances of barriers and facilitators to providing smoking cessation support can now be incorporated into our a-priori proposition:

> In sites with fewer barriers and more facilitators to providing cessation support, more women will stop smoking (primary outcome), engage with SSS, be recruited to and retained in the trial. This would indicate that maternity and SSS potentially provide an influential contribution to the offer of financial incentives which could inform implementation decisions.

The counter-factual null hypothesis would be:

> Financial incentives are effective regardless of the differing characteristics, barriers and facilitators to the provision of SSS support and usual care.

The numbers of participants taking part in individual CPIT III sites do not provide the statistical power to draw conclusions about the effectiveness of financial incentives on a local basis. However, further exploratory descriptive analysis of CPIT III results by trial site will now be undertaken by the trial statistician and health economists working independently of the process evaluation team. How usual care and trial outcomes vary across sites will be important for understanding generalisability for implementation of financial incentives.

For peer review only - http://bmjopen.bmj.com/site/about/guidelines.xhtml

AUTHOR CONTRIBUTIONS

Jennifer McKell led the data collection, analysis and reporting relating to the process evaluation. JM is registered for a part time PhD by publication at the University of Stirling which is based on the CPIT III process evaluation. Pat Hoddinott and Fiona M. Harris designed the process evaluation for the original grant application, oversaw data collection and analysis and are PhD supervisors. Lesley Sinclair was the trial manager of CPIT III and David Tappin and Linda Bauld were co-principal investigators of CPIT III and designed the overall study. All authors contributed to drafting and reviewing the manuscript

ACKNOWLEDGEMENTS

The authors wish to thank service users, trial participants, maternity and smoking cessation staff who supported the process evaluation by taking part in formal or informal interviews, permitting observation of usual care or facilitating data collection within case study sites. Our thanks also to trial staff who provided their perspectives on trial progress locally. Finally, our thanks to Dr. Isabelle Uny, Research Fellow in the Institute for Social Marketing and Health at the University of Stirling, for her assistance with early process evaluation data collection and analysis.

COMPETING INTERESTS

The authors declare no competing interests

FUNDING

This study was supported by the Chief Scientist Office of the Scottish Government, grant number HIPS/16/01/CSO; with partnership funding from Cancer Research UK, grant number C48006/A20863/CRUK; Health and Social Care Northern Ireland COM/5352/17; Northern Ireland Chest Heart & Stroke 2017_09; Health and Social Care Northern Ireland Public Health Agency (no grant number); Lullaby Trust P272; Scottish Cot Death Trust (no grant number).

DATA SHARING

Data sharing is not possible for this study as participants were not asked for permission to share their data to avoid deterring participation, particularly from professionals working in services that may be identifiable.

ETHICS APPROVAL STATEMENT

Ethical approval for CPIT III, including the process evaluation, was given by the West of Scotland Research Ethics Committee 4, reference: 17/WS/0173.

For peer review only - http://bmjopen.bmj.com/site/about/guidelines.xhtml

BIBLIOGRAPHY

1. The Health Consequences of Smoking-50 Years of Progress: A Report of the Surgeon General. Atlanta (GA): Centers for Disease Control and Prevention (US) 2014.
2. Doll R, Peto R, Boreham J, et al. Mortality in relation to smoking: 50 years' observations on male British doctors. *BMJ* 2004;328(7455):1519. doi: 10.1136/bmj.38142.554479.AE [published Online First: 2004/06/24]
3. Notley C, Gentry S, Livingstone-Banks J, et al. Incentives for smoking cessation. *Cochrane Database Syst Rev* 2019;7:CD004307. doi: 10.1002/14651858.CD004307.pub6 [published Online First: 2019/07/18]
4. National Institute for Health and Care Excellence (NICE). Tobacco: preventing uptake promoting quitting and treating dependence [NG209]. London: NICE, 2021.
5. Pawson R, Tilley N. Realistic evaluation. London: SAGE 1997.
6. Morgan H, Hoddinott P, Thomson G, et al. Benefits of Incentives for Breastfeeding and Smoking cessation in pregnancy (BIBS): a mixed-methods study to inform trial design. *Health Technology Assessment (Winchester, England)* 2015;19(30):1-522, vii.
7. Radley A, Ballard P, Eadie D, et al. Give It Up For Baby: outcomes and factors influencing uptake of a pilot smoking cessation incentive scheme for pregnant women. *BMC Public Health* 2013;13:343. doi: 10.1186/1471-2458-13-343 [published Online First: 2013/04/17]
8. Mantzari E, Vogt F, Marteau TM. The effectiveness of financial incentives for smoking cessation during pregnancy: is it from being paid or from the extra aid? *Bmc Pregnancy and Childbirth* 2012;12 doi: 10.1186/1471-2393-12-24
9. National Institute for Health and Care Excellence (NICE). Smoking: stopping in pregnancy and after childbirth [PH26]. London: NICE, 2010.
10. Tappin D, Bauld L, Purves D, et al. Financial incentives for smoking cessation in pregnancy: randomised controlled trial. *BMJ* 2015;350:h134. doi: 10.1136/bmj.h134 [published Online First: 2015/01/30]
11. Sinclair L, McFadden M, Tilbrook H, et al. The smoking cessation in pregnancy incentives trial (CPIT): study protocol for a phase III randomised controlled trial. *Trials* 2020;21(1):183. doi: 10.1186/s13063-019-4042-8 [published Online First: 2020/02/16]
12. Yin RK. Case study research : design and methods. 2nd ed. ed. Thousand Oaks, Calif. ; London: Sage 1994.
13. Stake RE. The art of case study research. Thousand Oaks ; London: Sage Publications 1995.
14. Ritchie J, Lewis J. Qualitative research practice : a guide for social science students and researchers. London: SAGE 2003.
15. Gale NK, Heath G, Cameron E, et al. Using the framework method for the analysis of qualitative data in multi-disciplinary health research. *BMC medical research methodology* 2013;13(1):1-8.
16. Moore L, Campbell R, Whelan A, et al. Self help smoking cessation in pregnancy: cluster randomised controlled trial. *Bmj* 2002;325(7377):1383.
17. Bell R, Glinianaia SV, van der Waal Z, et al. Evaluation of a complex healthcare intervention to increase smoking cessation in pregnant women: interrupted time series analysis with economic evaluation. *Tobacco control* 2018;27(1):90-98.
18. Jones S, Hamilton S, Bell R, et al. What helped and hindered implementation of an intervention package to reduce smoking in pregnancy: process evaluation guided by normalization process theory. *BMC health services research* 2019;19(1):1-13.
19. Jones SE, Hamilton S, Bell R, et al. Acceptability of a cessation intervention for pregnant smokers: a qualitative study guided by Normalization Process Theory. *BMC public health* 2020;20(1):1-10.

For peer review only - http://bmjopen.bmj.com/site/about/guidelines.xhtml

20. Bryce A, Butler C, Gnich W, et al. CATCH: development of a home-based midwifery intervention to support young pregnant smokers to quit. *Midwifery* 2009;25(5):473-82.

For peer review only

For peer review only - http://bmjopen.bmj.com/site/about/guidelines.xhtml

CPIT 3 Process Evaluation

Nodes\\Revised Coding Framework

Coding Framework revised after testing by JM and IU August 2019

Name	Description
01, Job Role, professional background and experience	
a) Description of job role and relevant training	Including length of time in current post and professional background
b) Role within the trial	Including feelings about role within the trial
e) Professional standards or codes of conduct	
f) Importance of research evidence to role or practice	
02, Trial introduction and set-up	
a) How and when first heard about trial and from whom	
b) Perspectives of the trial team	

For peer review only - http://bmjopen.bmj.com/site/about/guidelines.xhtml

Name	Description
c) Initial thoughts and feelings about using incentives to encourage smoking cessation	Including interest in the trial
d) Initial thoughts and feelings about service or employer being involved with trial	
e) Decision-making about involvement in the trial	
f) Expectations of the trial	Including thoughts on execution of the research - also thoughts on CPIT phase 2 results
g) Preparations and training for CPIT	
03, Routine Service Characteristics	
a) Nature of booking appointment and midwifery care generally	Including impact of national policies on midwifery care.
b) Discussion of smoking within booking appointments	Where relevant to discussion of smoking also mention of CO monitoring
c) CO monitoring	
d) Criteria for and method of referral plus how women are first contacted by SSS	Also includes the typical number of referrals received each month by the service

For peer review only - http://bmjopen.bmj.com/site/about/guidelines.xhtml

Name	Description
e) Approach to and nature of support from smoking cessation service	Including level of engagement from women (prior to the trial). Also includes approach of midwives to support e.g specialist substance use midwives
f) Access to NRT and ability to prescribe directly to clients	
g) Monitoring of referral, engagement and outcomes	
h) Links between maternity staff and SSS	Including perspectives of the other group
i) Description and history of service or employing organisation	Also, description of management structures and organisational change prior to the trial. Description of how they feel about service including possible ownership. Also relationship with women - level of engagement
j) Discussion of local or national strategies with relevance to service or individual role	
04, Delivery and process of trial (feasibility)	
a) Introducing the trial to women referred	Including checking for eligibility
b) Consent call from ECHO	Including thoughts on randomisation to one of two groups

For peer review only - http://bmjopen.bmj.com/site/about/guidelines.xhtml

Name	Description
c) Face to face contact with adviser and setting a quit date	
d) Smoking status phone calls or research nurse visits for CO monitoring and triggering of incentives	
e) End of pregnancy smoking status, CO monitoring and cotinine testing	
f) Final smoking status check at 6 months post partum	
g) Client receiving or using financial incentives at key stages, practical issues	
h) Timing issues and any impact of delays	
05, Impact of the trial on services and women	
a) Maternity Service	
b) Smoking Cessation Service	
c) Women	

For peer review only - http://bmjopen.bmj.com/site/about/guidelines.xhtml

Name	Description
06, Making the trial work	
a) Information sharing and communication between trial staff and SSS	
b) Cooperation and compromise	
c) Support from the trial to the SSS	
07, Incentives and motivation	
a) Perspectives of acceptability of using incentives to promote smoking cessation in pregnancy	
i) Any awareness of incentives being used to change other behaviours	
ii) Feelings regarding the use of incentives	
iii) Type of incentive i.e financial versus other	
iv) Amounts and staged approach	

For peer review only - http://bmjopen.bmj.com/site/about/guidelines.xhtml

Name	Description
v) Thoughts on possible impact including length of time offered	
b) Perceived impact on client motivation to engage, to quit, to stay quit	
i) Overview of motivating factors for quit	
ii) Perceived impact of incentives	Including extent to which there has been greater engagement from women or the types of women who are accessing support
iii) Relative importance of incentives in context of other intervention factors	e.g. Role of smoking cessation adviser or CO monitoring
iv) Relative importance of incentives in context of other personal factors	e.g health, saving money, stress, smoking by partner or family and friends
v) Women's feedback on incentives	Including what incentives have been used on
vi) Any impact on professional appreciation of using incentives	i.e as a selling point

For peer review only - http://bmjopen.bmj.com/site/about/guidelines.xhtml

Name	Description
08, Local context and characteristics of population served by smoking cessation service	
a) Geography in terms of rural or urban setting	
b) Socio-economic background	
c) Age	
d) Ethnic mix	
f) Other important characteristics of local population	
09, Recommendations for improving the trial and learning for future rollout	
a) Recommendations to improve recruitment to the trial	
b) Recommendations to change type, amount or timing of incentives	
c) Recommendations to improve follow up of participants including monitoring	

For peer review only - http://bmjopen.bmj.com/site/about/guidelines.xhtml

Name	Description
d) Learning useful for future rollout from perspective of participants	
e) Learning useful for future rollout from perspective of smoking cessation service	
f) Views or suggestions for the process evaluation	
g) Learning points for running a trial within a service or organisation	
h) What worked in Glasgow and comparison with current sites	
10, Impression of Interview	Manner and tone of participant
11 Miscellaneous	
12 Barriers to Quitting	
13, Experience of supporting pregnant women to stop smoking*	
14, Progress of trial*	
14.1 Changes to the trial process	

For peer review only - http://bmjopen.bmj.com/site/about/guidelines.xhtml

Name	Description
15, Working with (Name of call centre company managing trial consent process)*	

* New nodes added by JM after revised framework was finalised in August 2019

For peer review only

For peer review only - http://bmjopen.bmj.com/site/about/guidelines.xhtml

COREQ Checklist: ***Usual care in a multi-centre randomised controlled trial of financial incentives for smoking cessation in pregnancy: qualitative findings from a mixed methods process evaluation***

No	Item	Presence (Y/N)	Page Number
Domain 1: Research Team and reflexivity			
Personal Characteristics			
1	Interviewer/facilitator	Y	6
2	Credentials	Y	14
3	Occupation	N	N/A
4	Gender	Y	6
5	Experience and training	Y	6
Relationships with participants			
6	Relationship established	N	N/A
7	Participant knowledge of the interviewer	N	N/A
8	Interviewer characteristics	N	N/A
Domain 2: Study Design			
Theoretical framework			
9	Methodological orientation and theory	Y	4 and 6
Participant selection			
10	Sampling	Y	6
11	Method of approach	N	N/A
12	Sample size	Y	5
13	Participation	N	N/A
Setting			
14	Setting of data collection	N	N/A
15	Presence of non-participants	N	N/A
16	Description of sample	N	N/A
Data Collection			
17	Interview guide	N	N/A
18	Repeat interviews	N	N/A
19	Audio-visual recording	N	N/A
20	Field notes	N	N/A
21	Duration	Y	6
22	Data saturation	N	N/A
23	Transcripts returned	N	N/A
Domain 3: Analysis and Findings			
Data Analysis			
24	Number of data coders	Y	6
25	Description of coding tree	Y	6 (supplementary file)
26	Derivation of themes	N	N/A
27	Software	Y	6
28	Participant checking	N	N/A
Reporting			
29	Quotations presented	Y	8-11
30	Data and findings consistent	Y	7-12
31	Clarity of major themes	Y	7, 8
32	Clarity of minor themes	Y	8-11

For peer review only

BMJ Open

Usual care in a multi-centre randomised controlled trial of financial incentives for smoking cessation in pregnancy: qualitative findings from a mixed-methods process evaluation

Journal:	*BMJ Open*
Manuscript ID	bmjopen-2022-066494.R1
Article Type:	Original research
Date Submitted by the Author:	26-Oct-2022
Complete List of Authors:	McKell, Jennifer; University of Stirling<br>Harris, Fiona M.; University of the West of Scotland<br>Sinclair, Lesley; University of York, Department of Health Sciences<br>Bauld, Linda; University of Edinburgh, Usher Institute of Population Health Sciences and Informatics<br>Tappin, David; University of Glasgow, Child Health<br>Hoddinott, Pat; University of Stirling, Nursing, Midwifery and Allied Health Professional Research Unit
**Primary Subject Heading**:	Smoking and tobacco
Secondary Subject Heading:	Qualitative research, Health services research, Research methods
Keywords:	QUALITATIVE RESEARCH, PUBLIC HEALTH, Maternal medicine < OBSTETRICS

SCHOLARONE™
Manuscripts

I, the Submitting Author has the right to grant and does grant on behalf of all authors of the Work (as defined in the below author licence), an exclusive licence and/or a non-exclusive licence for contributions from authors who are: i) UK Crown employees; ii) where BMJ has agreed a CC-BY licence shall apply, and/or iii) in accordance with the terms applicable for US Federal Government officers or employees acting as part of their official duties; on a worldwide, perpetual, irrevocable, royalty-free basis to BMJ Publishing Group Ltd ("BMJ") its licensees and where the relevant Journal is co-owned by BMJ to the co-owners of the Journal, to publish the Work in this journal and any other BMJ products and to exploit all rights, as set out in our licence.

The Submitting Author accepts and understands that any supply made under these terms is made by BMJ to the Submitting Author unless you are acting as an employee on behalf of your employer or a postgraduate student of an affiliated institution which is paying any applicable article publishing charge ("APC") for Open Access articles. Where the Submitting Author wishes to make the Work available on an Open Access basis (and intends to pay the relevant APC), the terms of reuse of such Open Access shall be governed by a Creative Commons licence – details of these licences and which Creative Commons licence will apply to this Work are set out in our licence referred to above.

Other than as permitted in any relevant BMJ Author's Self Archiving Policies, I confirm this Work has not been accepted for publication elsewhere, is not being considered for publication elsewhere and does not duplicate material already published. I confirm all authors consent to publication of this Work and authorise the granting of this licence.

For peer review only

Usual care in a multi-centre randomised controlled trial of financial incentives for smoking cessation in pregnancy: qualitative findings from a mixed-methods process evaluation

Jennifer McKell[1], Fiona M. Harris[2], Lesley Sinclair[3], Linda Bauld[4], David Tappin[5], Pat Hoddinott[1]

[1]University of Stirling, Stirling, UK

[2]University of the West of Scotland, Paisley, UK

[3]University of York, York, UK

[4]University of Edinburgh and SPECTRUM Research Consortium, Edinburgh, UK

[5]University of Glasgow, Glasgow, UK

Correspondence to:

Jennifer McKell

Institute for Social Marketing and Health, University of Stirling, Stirling, FK9 4LA

j.e.mckell@stir.ac.uk

For peer review only

ABSTRACT

Objectives

Financial incentives are recommended by the UK's National Institute for Health and Care Excellence (NICE) to aid smoking cessation in pregnancy. However, little is known about how implementation contexts might impact on their effectiveness. Variations in smoking cessation support (usual care) for pregnant women who smoke were examined qualitatively as part of a prospective process evaluation of the Cessation in Pregnancy Incentives Trial (CPIT III).

Design

Longitudinal case studies of five CPIT III trial sites informed by realist evaluation.

Setting

A stop smoking service (SSS) serving a maternity hospital constituted each case study, located in three UK countries.

Participants

Data collection included semi-structured interviews with trial participants (n=22), maternity (n=12) and SSS staff (n=17); and site observations and perspectives recorded in fieldnotes (n=85).

Results

Cessation support (usual care) for pregnant women varied in amount, location, staff capacity, flexibility and content across sites. SSS staff capacity was important to avoid gaps in support. Co-

For peer review only - http://bmjopen.bmj.com/site/about/guidelines.xhtml

location and good working relationships between maternity and SSS professionals enabled prioritisation and reinforced the importance of smoking cessation. Sites with limited use of carbon monoxide monitoring reduced opportunities to identify smokers while inconsistency around automatic referral processes prevented the offer of cessation support. SSS professionals co-located within antenatal clinics were available to women they could not otherwise reach. Flexibility around location, timing and tailoring of approaches for support, facilitated initial and sustained engagement, and reduced the burden on women.

Conclusions

Trial sites faced varied barriers and facilitators to delivering cessation support, reflecting heterogeneity in usual care. If financial incentives are more effective with concurrent smoking cessation support, sites with fewer barriers and more facilitators regarding this support would be expected to have more promising trial outcomes. Future reporting of trial outcomes will assist in understanding incentives' generalisability across a wide range of usual care settings.

Trial registration number: ISRCTN15236311.

STRENGTHS AND LIMITATIONS OF THIS STUDY

- This study uniquely examines, in depth, usual care provided during a multi-centre trial of financial incentives for smoking cessation in pregnancy
- Observations of usual care delivery, and environment, plus interviews with trial participants and health care staff allowed analysis of multiple perspectives on usual care and trial impact.
- Anonymous presentation of case study data allows for a more candid discussion of barriers and facilitators within sites.
- Case study sites represented populations with a majority white British background therefore barriers and facilitators to cessation support may differ in other more diverse populations.
- Barriers and facilitators relating to communication skills, such as motivational techniques used by SSS professionals to support cessation in pregnant women, were not considered.

INTRODUCTION

Smoking in pregnancy raises the risk of serious consequences for mothers and babies, including miscarriage, still birth, prematurity, birth defects, and infant death[1]. Women who continue to smoke can lose on average 10 years of life whereas those who give up permanently by age 40 during their childbearing years are likely to have a normal lifespan[2]. Identifying effective and cost-effective ways to help more women to quit smoking during pregnancy remains vital, and evidence supports the use of financial incentives[3]. In 2021, NICE recommended the use of financial incentives to help pregnant women to quit smoking[4].

Alongside effectiveness and cost effectiveness for behavioural interventions it is important to understand how complex interventions work[5]. Support received by pregnant women alongside incentives may be a factor influencing effectiveness[6]. A pilot study in Scotland found that participants most successful in their quit attempts were those who perceived financial incentives as

For peer review only - http://bmjopen.bmj.com/site/about/guidelines.xhtml

'part of a wider reward structure' involving regular engagement with specialist support[7]. Similarly, Mantzari et al (2012) found that cessation support held greater importance for incentivised compared to non-incentivised women, with the former particularly motivated by 'being monitored'[8]. This evidence followed the publication of NICE guidelines in 2010, recommending 'intensive and ongoing support' of pregnant women by National Health Service (NHS) smoking cessation services as part of usual care[9]. All pregnant women resident in the UK are eligible to receive free maternity care provided by the NHS. There are differences in how care is provided across the devolved UK nations[10-12] but generally this involves a combination of hospital and community-based care. Smoking cessation support within pregnancy, in the UK, varies from one area to the next but particular characteristics occur regularly[13].

Offering smoking cessation support alongside financial incentives was a key characteristic of the single centre Cessation in Pregnancy Incentives Trial (CPIT II)[14]. Participants receiving the addition of incentives were two and a half times more likely to be abstinent at the end of pregnancy compared with SSS support alone (22.5%, 69/306; versus 8.6%, 26/303). The CPIT III trial commenced in 2018, replicating the CPIT II intervention and trial design in seven sites across three UK countries. CPIT III aimed to establish, the effectiveness, cost effectiveness and generalisability of adding the offer of financial incentives to usual care to stop smoking during pregnancy[13]. Like the findings of CPIT II, CPIT III found that participants receiving incentives in addition to usual care were more than twice as likely to be smoke free in late pregnancy compared to participants receiving usual care alone (26.8%, 126/471; versus 12.3%, 58/470)[15].

The trial protocol[13] included a mixed-methods process evaluation to identify and understand differences in usual care for pregnant smokers; the geographic and population mix of trial sites and their relationship to trial conduct and outcomes.

This paper sets out the barriers and facilitators to providing SSS (usual care) at five of seven trial sites. An a-priori proposition underpinned the process evaluation:

> Sites would vary in the usual care provided for pregnant women who smoke and therefore trial outcomes would vary by site, indicating that maternity and SSS interact with the offer of financial incentives.

METHODS

Process evaluation design

A longitudinal, mixed-methods case study design, informed by realist evaluation, formed the basis of the embedded process evaluation[5 16 17]. The case studies, defined as a trial site (an SSS serving a maternity hospital), included 5 of the 7 trial sites representing diverse service configurations. Being one of the first five sites to open to recruitment was the criteria for selection of case study sites, though the fifth site chosen opened later than expected. All case study sites were selected independent of the main trial team. Methods and findings described in this paper relate to qualitative examination of usual care. Further details on the design of the CPIT III full process evaluation are reported elsewhere[13].

Patient and public involvement

Two CPIT II participants and members of the UK Centre for Tobacco and Alcohol Studies (UKCTAS) smokers' panel were involved in planning CPIT III; and a patient representative was included in the

For peer review only - http://bmjopen.bmj.com/site/about/guidelines.xhtml

CPIT III trial steering committee. Resources were not available to provide separate patient and public involvement in the process evaluation and the onset of the Covid-19 pandemic towards the end of the study, and during analytical and interpretative stages, further restricted opportunities for further involvement.

Data collection

Case study data collection occurred in 2 stages. Stage 1 (October 2017 to December 2019) established the context for the trial locally via observation of usual care of pregnant smokers and informal interviews with maternity or SSS staff recorded as fieldnotes. Stage 2 (July 2018 to September 2020) involved qualitative, semi-structured, interviews using pre-determined topic guides (supplementary files 1 and 2) with trial participants, maternity and SSS staff (health care staff) to explore experiences and perceptions relating to the trial and usual care. Concurrent to stages 1 and 2, the process evaluation team recorded, also in fieldnotes, trial staff perspectives relating to case study sites from trial management working group (TMWG) (n=31) meetings and informal discussions with the trial manager (n=6) and site-based trial staff (n=16). Case study specific data sources are detailed in Table 1. To protect the anonymity of interviewees and informants case study sites are referred to as A-E.

Table 1: Case study specific data sources (n=5)

Data source	Site A	Site B	Site C	Site D	Site E	Total
Observations of maternity booking or other early ante-natal appointments	2	3[&]	2	5	3	15
Observations of smoking cessation consultations	3	3	2	2	2	12
Informal interviews with maternity or SSS staff	1	1	1	1	1	5
Interviews with maternity staff	2	4[#]	3	3	0^	12
Interviews with SSS staff	3[#]	3	3	5	3	17
Interviews with trial participants	2	4*	6	5	5	22

For peer review only - http://bmjopen.bmj.com/site/about/guidelines.xhtml

Informal discussions with local trial team members (number of trial team members consulted)	3(1)	2(1)	5[+](3)	5 (2)	1(1)	16
Total	16	20	22	26	15	99

[&]Includes observation of a training session for midwives delivered by SSS

^It was not possible to conduct interviews with maternity staff due to the onset of the Covid-19 pandemic and ongoing pressures up to the end of qualitative data collection in December 2020;

[#] One interview included two interviewees

*Includes 2 interviewees who indicated an interest in the study but were not randomised;

+Includes one semi-structured formal interview with a local research lead, conducted by telephone. Two other trial team members consulted 4 times.

For peer review only

Data collection was undertaken by JM with assistance from IU (please see Acknowledgements) who are both female qualitative researchers with experience of research into smoking cessation and pregnant women. Interviews with trial participants were conducted by telephone and lasted on average approximately 20 minutes, with duration ranging from 9 to 36 minutes. Sample sizes at sites were proportionate to the numbers recruited and aimed to achieve maximum diversity in participant age, ethnicity and socio-economic characteristics in sites with higher numbers. Two women who consented to contact about the trial but did not consent to be randomised were interviewed but elicited little additional data. Interviews with health care staff, were conducted either face to face (n=19) or via telephone (n=10) and ranged from 11 to 57 minutes in duration, or on average 30 minutes. Face-to-face interviews were conducted in workplaces (apart from one in a public place). Average duration of face-to-face interviews was similar to that of telephone interviews, at around 36 minutes. Two, face-to-face health care staff interviews involved the participation of two interviewees in each; the remaining interviews had no other people present. All interview participants were unknown to researchers prior to study commencement, and information provided before interviews explained the researcher's role in the research.

Analysis

Analysis of data drew on the Framework method[18 19] informed by realist evaluation. Transcripts of interviews with health care staff and field notes from site observations, informal interviews with health care staff, discussions with trial staff and trial meetings were uploaded to QSR Nvivo (v12) software for purposes of organisation and management. Informed by themes in the topic guides but also themes arising from the data, an initial coding framework was created, tested and developed by JM and IU to produce a finalised version (supplementary file 3). This was then applied to the data in the Nvivo file as part of the indexing stage. Transcripts of trial participant interviews were read and indexed separately according to two themes: perspectives of usual care and trial processes, respectively. As part of the charting phase, matrices were developed for each case study site

For peer review only - http://bmjopen.bmj.com/site/about/guidelines.xhtml

containing data summaries incorporating the theme of 'routine service characteristics' from the coding framework and perspectives of usual care from trial participant interviews. Barriers and facilitators to cessation support (usual care) were then drawn out by comparing the data summaries to identify similarities and contradictions in the data. Identified barriers and facilitators were considered in depth to establish Context, Mechanism Outcome configurations (CMO)

CMOs were developed with input from the wider process evaluation team (PH and FH), which included both social science and clinical input. The 'a priori' proposition was then revisited in the light of our findings, to inform the discussion.

Participant confidentiality was important to facilitate honest and information-rich accounts of usual care. Staff job titles within SSS significantly varied across the sites, often reflecting their occupations. In some sites, those providing cessation support were specialist midwives, while in another, nurses delivered cessation support. In two sites, support was delivered by 'facilitators' or 'wellbeing workers', with no clinical background. To minimise jigsaw identification of people or sites, quotes from SSS staff that deliver cessation support are referred to using the generic term 'SSS professional', regardless of occupational background, and 'senior' used for individuals with line management responsibility. Any potentially identifying information has been removed.

RESULTS

Usual care settings varied considerably (Table 2). Barriers and facilitators to delivering SSS support during a financial incentives trial are then presented, with CMO configurations to highlight the implications for usual care.

Table 2: Usual care settings in CPIT III trial sites

	Site A	Site B	Site C	Site D	Site E
Policy of referral (opt-in or opt-out)	Opt-out	Opt-out	Opt-out	Opt-out	Opt-out
Target population and behaviour change supported	Pregnant women, smoking and weight management	Pregnant women, smoking only	Pregnant women, smoking only	General population, smoking only	General population, smoking, alcohol, weight management and physical activity
Organisation providing SSS*	NHS	NHS	NHS	NHS	Local Government
SSS professionals with or without a midwifery/ nursing background	Mixed – some with and some without a midwifery/ nursing background	Midwifery/ nursing background	Midwifery/ nursing background	Midwifery/ nursing background	No midwifery/ nursing background

For peer review only - http://bmjopen.bmj.com/site/about/guidelines.xhtml

SSS funder (and location of line management)	Local Government (NHS Trust/Board)	NHS Trust/Board (NHS Trust/Board)	Public Health Body (NHS Health Improvement)	NHS Health Improvement (NHS Health Improvement)	Local Government (Local Government)
Venue for SSS consultations	Hospital consultation room or other space (not dedicated). Also, a GP Surgery room for those remotely located	Hospital consultation room (dedicated) or home visit	Hospital consultation room (dedicated)	Community venue (regular drop-in or by appointment) or home visit	Community venue (regular drop-in or by appointment)
Methods of SSS consultation	Face-to-face or telephone	Face-to-face or telephone	Face-to-face or telephone	Face-to-face or telephone	Face-to-face or telephone
SSS ability to provide NRT directly	No	Yes	No	Yes	No
Co-location of SSS and maternity services	Yes	Yes	Yes	No (in most cases)	No

*SSS has been used to denote stop smoking services within trial sites, although some were not necessarily known as such, as they provided wider behaviour change services.

Barriers and facilitators to providing usual care in trial sites

Case studies highlighted barriers and facilitators to providing usual care, particularly in relation to professional relationships and service capacity. Barriers and facilitators were also identified in maternity services' ability to identify smoking in pregnancy and SSS ability to engage women in cessation support, as well as features of that support, as described below. Each section concludes with the CMO configuration.

SSS capacity

Limitations on SSS staff capacity (number, workload, absence cover, unfilled vacancies) in trial sites affected cessation support. In site C, capacity issues arose during periods of long term leave among key staff in a small team who were not quickly replaced or on the same number of hours. An intervention participant noted the impact of this staff shortage saying she would have liked more support from the study team.

> *I wasn't getting enough phone calls I don't think, […], obviously because I wasn't getting the [SSS professional] support I would have liked you know a wee phone call just, not like a formal phone call to say oh you are going to get a voucher, but just like a wee phone call to*

For peer review only - http://bmjopen.bmj.com/site/about/guidelines.xhtml

say "oh how are you doing?;[…]", I know that's not like your job or whatever but I think that would have made it a wee bit more easier (Intervention participant, Site C)

The larger SSS in site D experienced numerous staff leaving during the study period and slow recruitment to posts. However, these difficulties were not reflected in participant or SSS professionals' perspectives. Yet evidence suggested that the service may be struggling with capacity. Trial staff noticed increasing numbers of participants being referred to local pharmacies for support, plus inconsistent CO monitoring, as per the CPIT III trial protocol[13], in intervention participants to verify smoking abstinence 4 weeks after setting a quit date.

Staff capacity issues were less prominent in other sites but a SSS manager in site E (supporting multiple behaviour change) noted that staff numbers had reduced by more than half despite increasing demand:

[name of SSS] […] at the time, I think, had 41.5 full-time equivalent employees. So, we're talking about a vast big service. I think it was like £1.1 million pounds the service cost and now I think we've got 16.5 full-time equivalents, (…). So, we've slowly got smaller and smaller, and I would say that the demand on our service is far, far, higher than it ever was when we were 41.5 equivalent employees. (Senior SSS professional, Site E)

Problems with capacity in SSS (C) reduced the time and attention staff could provide (M), increasing the likelihood of sub-optimal cessation support for pregnant women (O).

Communication and connections between midwives and SSS professionals

Shared working environments and positive, constructive relations between maternity and SSS were beneficial. Midwives and SSS professionals in three sites were co-located in the same hospital, often within easy reach, and such proximity allowed development of close, amiable working relationships. In site B, the SSS was embedded within the maternity unit, as demonstrated by efforts to extend CO monitoring in midwife appointments:

…when we brought in carbon monoxide and every opportunity we see them … everyone [midwives] initially thinks: 'oh my God that is extra work' and we go: 'well you are already doing their blood pressure and dipping their urine so by the time you've done that, you know, it won't take you that much longer', and everyone has just got used to it… they sort of, are used to us saying oh we are doing this now and explaining it… (SSS professional, Site B)

Proximity working, however, was no guarantee of good working relationships. Midwives and SSS staff in one site shared consultation rooms and corridors, but gaps existed between them in relation to approaches to smoking cessation, joint working, and communication. A senior SSS staff member highlighted mismatched priorities between maternity and SSS staff.

Community midwives what they say…yes there are some that are still perhaps not presenting it in the way that would benefit (…) the women are saying 'oh no I don't want to quit. I don't want to see them' and rather than perhaps being more positive about the [SSS] service and saying they'll have a good chat with you about the benefits of quitting or whatever. They are just informing us that these women don't want to be approached. (Senior SSS professional, Site A)

In two sites, maternity and SSS staff never or only occasionally shared the same work site, despite both working within community settings. The extent of links between staff groupings was sufficient

For peer review only - http://bmjopen.bmj.com/site/about/guidelines.xhtml

for most but one SSS professional said they would like midwives' support to engage pregnant smokers.

> *if we could go and see them [midwives] and say look that girl hasn't turned up and she's coming back, if we can't get her on the phone, it might be helpful if they speak to them then. (…) So that they're getting the advice from, at each contact basically (…)* (SSS professional, Site D)

Co-location and good working relationships between maternity and smoking cessation professionals (C) prioritises and reinforces smoking cessation (M) optimising the importance of quitting for women (O).

Identifying pregnant smokers

Identification of smokers took place during initial appointments with midwives. Women were asked if they smoked and to provide a carbon monoxide (CO) breath test. This was requested of all pregnant women, and not only those who self-reported smoking, to detect CO exposure and provided further opportunity to identify smokers. All sites operated an opt-out referral policy which meant that pregnant women who self-reported as smokers/recent ex-smokers or exceeded a particular CO level were automatically referred to SSS.

The role and level of implementation of CO monitoring varied by site. There was clear expectation at some sites that midwives would undertake CO monitoring at every ante-natal appointment, whereas in others, CO monitoring only occurred at initial appointments. In most sites, exceeding a particular CO level, usually of 4 parts per million or above, triggered referral to SSS for both self-reported non-smokers and smokers. In contrast, at one site, self-reported non-smokers with high CO levels were not referred but recommended for future monitoring. This suggests the following CMO configuration:

Limitations on the role and use of CO monitoring within sites (C) reduced the opportunities for midwives to identify smoking in pregnancy (M) and in turn reduced opportunities to offer cessation support (O).

Differences in interpretation of automatic referral were evident among midwives, suggesting inconsistency in opportunities for smoking cessation support. Discussion of automatic, opt-out referral with staff across and within sites provided contrasting descriptions of the conditions for this. Perceptions included automatic referral not taking place if a woman particularly objected versus automatic referral conditional on specified criteria alone:

> *I haven't referred anybody against their will kind of thing, you know? We do try to treat it as more of a kind of opt out service so (…) So it's more they would need to kind of say, you know what I mean, instead of us just offering would you like this or not (Midwife, Site D)*

> *It's a mandatory referral at booking, (…) I always say you will get a phone call and it's their choice then as to whether they do, because we refer everybody. Sometimes a woman will say 'I am definitely not going to be engaging with them'. And then on the referral we will put (…) 'does not want to engage', so that's the end of it really but at least the team know about them (Midwife, Site B)*

This suggests: Adherence to automatic referral based on specified criteria (C), ensures that all women are approached and informed about availability of SSS (M) and raises the possibility of SSS engagement (O).

For peer review only - http://bmjopen.bmj.com/site/about/guidelines.xhtml

Engagement in cessation support

SSS used multiple methods to engage pregnant smokers following maternity referral, including phone-call, text and letter but cessation staff acknowledged that often women could not be reached or were lost to follow up. Access to ante-natal clinics, however, gave cessation staff an advantage in contacting women. Staff who worked within, or close to, ante-natal clinics benefitted from opportunities to approach women in person. However, there was no indication in the data that a face-to-face approach was important for establishing a continuing relationship.

> *…we are fortunate here in that we see the majority of women coming through the clinic either here or at one of the outlying clinics and for their initial scan. And so, if they're not picking the phone up to us then…we will more than likely see them when they attend for their scan and be able to have a discussion with them about smoking in pregnancy and whether they are ready to quit. (Senior SSS professional, Site A)*

Sites that enabled SSS professionals to approach women face to face at ante-natal clinics (C) provided a sense of immediacy for smoking cessation support and reduced the potential for avoidance (M), increasing the opportunities to offer support (O)

SSS ability to tailor the offer of support to optimise appeal to pregnant women also appeared important for engagement. In two sites, SSS saw opportunities in the way that smoking in pregnancy is discussed. Here staff advocated for initial conversations to emphasise exposure to CO rather than stopping smoking. A SSS professional in site C described a tailored approach for reluctant referrals.

> *it's just getting the wording right, (…) sometimes I would talk about you know, "you had your booking appointment the other day, how did you get on?, Was your scan okay?, great, I see one of your tests, your CO level was very high, it was into the red there, we'd be a bit worried there for your pregnancy, you are a smoker is that right?" (…) it's not like: [SSS professional]: "I am here to help you stop smoking"; [hypothetical woman]: "well, I don't want to stop smoking", hang up the phone." (SSS professional, Site C)*

At site D, SSS were unable to engage a trial participant allocated to incentives who declined support due to her perception of available support. The woman felt self-conscious of being a young pregnant smoker attending a pharmacy or a 'group': '*some people are too embarrassed to go to the groups*'; though the latter was an old SSS term for a drop-in session.

SSS' ability to customise their offer of cessation support (C) to maximise appeal to individuals with different perspectives and preferences (M) facilitated initial engagement with cessation support (O).

Features of cessation support

SSS' ability to offer flexibility regarding consultations at suitable times and convenient locations made it easier for women to engage. Participants spoke of the value of SSS provided near to where they lived or via home visits.

> *I can phone her [SSS professional] if I'm stuck at any point. But the good thing is as well is if there's a time that I'm unwell she can come out to me, I just phone her. (Intervention Group Participant, Site D)*

However, SSS were not always able to provide convenient access. A SSS located in a maternity unit did not have a dedicated consulting room and could only offer afternoon appointments after ante-

For peer review only - http://bmjopen.bmj.com/site/about/guidelines.xhtml

natal clinics. Travelling into the maternity unit was also a challenge for women living in more remote areas.

> *…certainly with the follow-ups because those appointments aren't quite so long, so women are quite reluctant to get on a bus for 45 minutes to have…to see one of us for 20 minutes. (Senior SSS professional, Site A)*

Site E's SSS was able to offer weekly drop-in sessions in a community centre, one of several service venues, but consultations were limited to 15 minutes. The service manager noted less flexibility over the location and timing of appointments if a longer one-to-one consultation was required.

Providing cessation support in accessible locations and/or at convenient times (C) facilitated engagement and minimised the commitment required from women (M) increasing opportunities for sustained engagement in support (O)

How nicotine replacement therapy (NRT) was provided also had an effect on outcomes. A lack of directly provided NRT in some SSS caused delays to quit attempts. While some SSS were able to provide NRT directly, others had to refer women to their GP or linked pharmacies for NRT.

> *So, we direct the email, the prescription request, to the doctor's surgery to guarantee it gets there. (…) We used to give them [women] it and they'd be like oh we didn't' make it to the chemist and they've still got it a week later and stuff like that. […] They can pick that up within forty-eight hours, but we do put urgent, so we can phone the doctors and ask if it can be quicker, if they want to try to get that quit date set quicker. (SSS professional, Site A)*

Provision of NRT at smoking cessation consultations (C) ensures immediate access to pharmacological support (M) enhancing pregnant smokers' ability to address nicotine withdrawal (O).

Some SSS showed a willingness to adapt to women's needs and preferences through innovation around support. Examples included extra support when dealing with low mood and depression; suggesting the use of e-cigarettes and introducing cessation support for significant others.

> *…we realised that one thing that we were coming up against quite a lot was problems with mental health and general wellbeing. So, we did an audit of our patients that were being referred into us and 50% to 60% of women that were being referred into our service had either mental health issues or general wellbeing issues […] we also run a class with [charity] […] So it all talks about mental health and antenatal depression, postnatal depression, things to look out for… (SSS professional, Site C)*

SSS willingness to innovate (C) extended the variety of support options available to women (M) increasing opportunities to identify effective cessation support (O).

DISCUSSION

Main findings

Cessation support provided as usual care by maternity services and SSS across trial sites varied substantially in terms of SSS staff capacity and relationships, how services identified pregnant women who smoke, and their ability to engage, support and help women to quit.

For peer review only - http://bmjopen.bmj.com/site/about/guidelines.xhtml

Limitations on staff capacity in particular sites affected the amount and type of cessation support available to women. Close contact and good relationships between midwives and SSS were generally beneficial, but in some sites, there was only limited contact and cohesiveness.

CO monitoring of pregnant women throughout pregnancy was standard procedure in some sites but not others and this limited identification. Automatic opt-out referral of women who were smoking or exceeded a CO threshold was official policy across all sites but differences in interpretation meant that some women who smoked were not referred. Working within or close to ante-natal clinics enabled SSS to approach women they may otherwise struggle to reach. SSS found benefit in tailoring their approach to take account of women's perspectives and preferences. Women valued cessation support consultations held in accessible locations at a convenient time of day, but some SSS were unable to offer this flexibility. Delays to women commencing quit attempts were identified when SSS could not provide NRT directly. Willingness and capacity by some sites to offer innovative approaches provided women with a greater variety of options and facilitated engagement with support.

Comparison of findings with other studies

This process evaluation is unique in detailing site variation in usual care within a randomised controlled trial of financial incentives for smoking cessation in pregnancy. Usual care was described as variable in a previous cluster randomised controlled trial of a self-help smoking cessation intervention for pregnant women, however no detail was provided, and the intervention was found to be ineffective[20]. An earlier systematic review of additional support provided alongside financial incentives for smoking cessation in pregnancy highlighted the potential importance of this as contributing to effectiveness[6]. An implementation study that examined the introduction of BabyClear©, an intervention involving universal CO monitoring in maternity booking appointments and an opt-out referral pathway, found that smoking cessation referrals and the proportion of women who had quit smoking by the time of birth in implementation sites increased[21]. A process evaluation identified barriers and facilitators to implementation that echoed findings in our study. As well as the benefits of partnership working between midwives and SSS and flexible appointments it also found inconsistency in CO monitoring[22 23]. In contrast to CPIT III, the authors identified midwives' reservations around discussing smoking with pregnant women. Other qualitative research similarly highlighted midwives' concerns about feeling poorly equipped to discuss smoking in pregnancy, but also differences among midwives in perspectives of opt-out referral, as discovered during CPIT III[24]. The same study also found barriers to good working relationships between midwives and SSS staff in terms of level of contact and prioritisation of smoking in pregnancy. A study of the development and implementation of an intervention to help young pregnant women to quit smoking found that home visits, sensitivity to inequalities, and direct provision of NRT were important facilitators to cessation[25]. The authors also considered support techniques provided by SSS and found motivational interviewing to be beneficial. Interestingly our findings did not uncover data to support a continuing relationship with a professional as important, although face-to-face opportunities did assist with initial engagement with SSS. This is consistent with findings of research aiming to prevent premature births, where increased continuity of care had no effect on smoking cessation as a secondary outcome[26].

Limitations

The study had a number of limitations. These included the predominance of the white British populations served by the trial sites, meaning that barriers and facilitators may differ for population groups not included in the study. There may have been issues with delivering behavioural support

For peer review only - http://bmjopen.bmj.com/site/about/guidelines.xhtml

when English is not the first language, or with differing cultural attitudes and behaviours related to smoking in pregnancy if a more diverse participant group had been included. In addition, resources did not permit more detailed data collection, observations and analysis of communication techniques regarding SSS communication with women.

Further work

CPIT III compared smoking cessation rates towards the end of pregnancy in women receiving usual care plus financial incentives versus usual care alone. However, with usual care among sites varying substantially, it raises the question 'what influence does this variation have on the effectiveness of financial incentives?'. Based on prior evidence that suggest cessation support could be a confounder for incentives[6], the findings of this process evaluation help to understand the generalisability of the overall CPIT III trial findings. This paper was completed prior to the research team analysing or knowing quantitative trial outcomes by site. This prospective exploratory process evaluation therefore minimises any bias that could arise from retrospective analysis once trial outcomes are known. The finding that CPIT III trial sites had differing characteristics and balances of barriers and facilitators to providing smoking cessation support can now be incorporated into our a-priori proposition:

> In sites with fewer barriers and more facilitators to providing cessation support, more women will stop smoking (primary outcome), engage with SSS, be recruited to and retained in the trial. This would indicate that maternity and SSS potentially provide an influential contribution to the offer of financial incentives which could inform implementation decisions.

The counter-factual null hypothesis would be:

> Financial incentives are effective regardless of the differing characteristics, barriers and facilitators to the provision of SSS support and usual care.

The numbers of participants taking part in individual CPIT III sites do not provide the statistical power to draw conclusions about the effectiveness of financial incentives on a local basis. However, further exploratory descriptive analysis of CPIT III results by trial site will now be conducted by the trial statistician and health economists working independently of the process evaluation team. How usual care and trial outcomes vary across sites will be important for understanding generalisability in the implementation of financial incentives.

** ** **

Contributors

Jennifer McKell (MSc) led the data collection, analysis and reporting relating to the process evaluation. JM is a Research Fellow at the University of Stirling and is registered for a part time PhD by publication which is based on the CPIT III process evaluation. Pat Hoddinott and Fiona M. Harris designed the process evaluation for the original grant application, oversaw data collection and analysis and are PhD supervisors. Lesley Sinclair was the trial manager of CPIT III and David Tappin

For peer review only - http://bmjopen.bmj.com/site/about/guidelines.xhtml

and Linda Bauld were co-principal investigators of CPIT III and designed the overall study. All authors contributed to drafting and reviewing the manuscript

Acknowledgements

The authors wish to thank service users, trial participants, maternity and smoking cessation staff who supported the process evaluation by taking part in formal or informal interviews, permitting observation of usual care or facilitating data collection within case study sites. Our thanks also to trial staff who provided their perspectives on trial progress locally. Finally, our thanks to Dr. Isabelle Uny (PhD), Research Fellow in the Institute for Social Marketing and Health at the University of Stirling, for her assistance with early process evaluation data collection and analysis.

Competing interests

The authors declare no competing interests.

Funding

This study was supported by the Chief Scientist Office of the Scottish Government, grant number HIPS/16/01/CSO; with partnership funding from Cancer Research UK, grant number C48006/A20863/CRUK; Health and Social Care Northern Ireland COM/5352/17; Northern Ireland Chest Heart & Stroke 2017_09; Health and Social Care Northern Ireland Public Health Agency (no grant number); Lullaby Trust P272; Scottish Cot Death Trust (no grant number).

Data availability statement

Data sharing is not possible for this study as participants were not asked for permission to share their data to avoid deterring participation, particularly from professionals working in services that may be identifiable.

Ethics approval

Ethical approval for CPIT III, including the process evaluation, was given by the West of Scotland Research Ethics Committee 4, reference: 17/WS/0173. Prior to semi-structured interviews with trial participants, and women who provided consent to contact about the trial but not consent to participate, researchers obtained informed consent based on the participant information sheet for the trial and verbal consent, audio-recorded and stored separately from interview recordings. Informed consent for semi-structured interviews with health care staff and a local research lead was obtained by provision of a study information sheet and a request to complete and sign an interview consent form.

For peer review only

References

1. The Health Consequences of Smoking-50 Years of Progress: A Report of the Surgeon General. Atlanta (GA): Centers for Disease Control and Prevention (US) 2014.
2. Doll R, Peto R, Boreham J, et al. Mortality in relation to smoking: 50 years' observations on male British doctors. *BMJ* 2004;328(7455):1519. doi: 10.1136/bmj.38142.554479.AE [published Online First: 2004/06/24]
3. Notley C, Gentry S, Livingstone-Banks J, et al. Incentives for smoking cessation. *Cochrane Database Syst Rev* 2019;7:CD004307. doi: 10.1002/14651858.CD004307.pub6 [published Online First: 2019/07/18]
4. National Institute for Health and Care Excellence (NICE). Tobacco: preventing uptake promoting quitting and treating dependence [NG209]. London: NICE, 2021.
5. Pawson R, Tilley N. Realistic evaluation. London: SAGE 1997.
6. Morgan H, Hoddinott P, Thomson G, et al. Benefits of Incentives for Breastfeeding and Smoking cessation in pregnancy (BIBS): a mixed-methods study to inform trial design. *Health Technology Assessment (Winchester, England)* 2015;19(30):1-522, vii.
7. Radley A, Ballard P, Eadie D, et al. Give It Up For Baby: outcomes and factors influencing uptake of a pilot smoking cessation incentive scheme for pregnant women. *BMC Public Health* 2013;13:343. doi: 10.1186/1471-2458-13-343 [published Online First: 2013/04/17]
8. Mantzari E, Vogt F, Marteau TM. The effectiveness of financial incentives for smoking cessation during pregnancy: is it from being paid or from the extra aid? *Bmc Pregnancy and Childbirth* 2012;12 doi: 10.1186/1471-2393-12-24
9. National Institute for Health and Care Excellence (NICE). Smoking: stopping in pregnancy and after childbirth [PH26]. London: NICE, 2010.
10. NHS. Your antenatal care: NHS; 2020 [updated 12/11/2020]. Available from: https://www.nhs.uk/pregnancy/your-pregnancy-care/your-antenatal-care/ accessed 07/10/2022.
11. NIdirect. Antenatal care [Available from: https://www.nidirect.gov.uk/articles/antenatal-care accessed 07/10/2022.
12. Public Health Scotland. Your antenatal care NHS Inform; [updated 08/04/2022]. Available from: https://www.nhsinform.scot/ready-steady-baby/pregnancy/your-antenatal-care/your-antenatal-care accessed 07/10/2022.
13. Sinclair L, McFadden M, Tilbrook H, et al. The smoking cessation in pregnancy incentives trial (CPIT): study protocol for a phase III randomised controlled trial. *Trials* 2020;21(1):183. doi: 10.1186/s13063-019-4042-8 [published Online First: 2020/02/16]
14. Tappin D, Bauld L, Purves D, et al. Financial incentives for smoking cessation in pregnancy: randomised controlled trial. *BMJ* 2015;350:h134. doi: 10.1136/bmj.h134 [published Online First: 2015/01/30]
15. Tappin D, Sinclair L, Kee F, et al. Effect of financial voucher incentives provided with UK stop smoking services on the cessation of smoking in pregnant women (CPIT III): pragmatic, multicentre, single blinded, phase 3, randomised controlled trial. *BMJ* 2022;379
16. Yin RK. Case study research : design and methods. 2nd ed. ed. Thousand Oaks, Calif. ; London: Sage 1994.
17. Stake RE. The art of case study research. Thousand Oaks ; London: Sage Publications 1995.
18. Ritchie J, Lewis J. Qualitative research practice : a guide for social science students and researchers. London: SAGE 2003.
19. Gale NK, Heath G, Cameron E, et al. Using the framework method for the analysis of qualitative data in multi-disciplinary health research. *BMC medical research methodology* 2013;13(1):1-8.
20. Moore L, Campbell R, Whelan A, et al. Self help smoking cessation in pregnancy: cluster randomised controlled trial. *Bmj* 2002;325(7377):1383.

For peer review only - http://bmjopen.bmj.com/site/about/guidelines.xhtml

21. Bell R, Glinianaia SV, van der Waal Z, et al. Evaluation of a complex healthcare intervention to increase smoking cessation in pregnant women: interrupted time series analysis with economic evaluation. *Tobacco control* 2018;27(1):90-98.
22. Jones S, Hamilton S, Bell R, et al. What helped and hindered implementation of an intervention package to reduce smoking in pregnancy: process evaluation guided by normalization process theory. *BMC health services research* 2019;19(1):1-13.
23. Jones SE, Hamilton S, Bell R, et al. Acceptability of a cessation intervention for pregnant smokers: a qualitative study guided by Normalization Process Theory. *BMC public health* 2020;20(1):1-10.
24. Naughton F, Hopewell S, Sinclair L, et al. Barriers and facilitators to smoking cessation in pregnancy and in the post-partum period: The health care professionals' perspective. *British journal of health psychology* 2018;23(3):741-57.
25. Bryce A, Butler C, Gnich W, et al. CATCH: development of a home-based midwifery intervention to support young pregnant smokers to quit. *Midwifery* 2009;25(5):473-82.
26. Fernandez Turienzo C, Bick D, Briley AL, et al. Midwifery continuity of care versus standard maternity care for women at increased risk of preterm birth: A hybrid implementation–effectiveness, randomised controlled pilot trial in the UK. *PLoS medicine* 2020;17(10):e1003350.

For peer review only

CPIT Qualitative Research Topic Guide
Interviews with Pregnant Women V1.0 05/07/2017

One-to-one telephone interviews with pregnant women presenting to the various study centres during the trial period, including women who decide to take part in the trial and those who decline.

Aim to explore views on issues around health professionals raising smoking in pregnancy i.e. midwives, referral to SSSs, discussion of study and decisions to take part or decline. Thoughts on consent phone call from NHS Stop smoking and random allocation to intervention or control.

Interviews will be adapted depending on experience, e.g. participation or non participation; intervention or control status,

The topic guide is intended to ensure coverage of key topics, whilst at the same time giving respondents the freedom to express their own feelings and views as part of an open discussion.

****

INTRODUCTION

- Provide a reminder of the purpose and main focus of the study
- Explain that focusing on opinions and experiences (not a test)
- Provide opportunity to ask questions
- Introduce the audio-recorder, underlining the importance of confidentiality
- Ask participant to complete consent form

A. BACKGROUND: BROAD SMOKING HISTORY AND LOCAL CONTEXT (ALSO WARM-UP QUESTIONS)

- How long smoked / how many a day / how easy to stop (anticipated/experienced)
- Any previous quit attempts/relapse events (inc. any previous pregnancies)
- When baby due
- How common is smoking where you live?
    - What do people you know think about smoking?

B. INTRODUCTION TO STOP SMOKING SUPPORT AND TRIAL PARTICIPATION

<u>Ante-natal appointment(s) with Midwife</u>
- Talk me through your first ante-natal visit
    - How did you feel about being advised to stop smoking?
    - How did you feel about referral to Stop Smoking Services (also where appropriate, automatic or opt-out referral)
    - How did you feel about CO monitoring (breath test)
- Any discussion of smoking at subsequent ante-natal appointments

<u>Initial contact(s) with NHS Smokefree Pregnancy Service Advisor</u>
- Explore telephone call OR drop-in at clinic
    - Thoughts on approach of advisor to stopping smoking
    - First thoughts when heard about the study from advisor
    - Everything clear/questions answered about trial
    - Reasons why you agreed/did not agree to having your contact details passed to the trial team

For peer review only

- Thoughts on information sheet received about trial: enough information; any questions

Trial Enrolment

- Can you talk me through what happened after you agreed to your details being passed to the trial team?
  - Thoughts and feelings about consent phonecall
- Why did you agree/decline to take part in the trial?
  - Were there any aspects of the trial that meant you were more or less likely to take part?
- Understanding of selection to intervention or control group
- Feelings about selection to intervention or control group
- Overall feelings about decision to take part after consent call

C. SUGGESTIONS FOR IMPROVING SERVICE AND/OR THE TRIAL

- Elements of the stop smoking service that are most/least useful – why?
- Things that might help more / improve the stop smoking service
- Promoting the incentive scheme
  - Key messages / ideas on 'selling points' for women
- Any thoughts on what would make it easier/more attractive for women to take part in the trial
- Anything else you would change or improve?

D. OVERVIEW

- Impact of being part of a trial, e.g. on motivation, impact of being a 'control'
- Any other/final thoughts?

For peer review only

CPIT Qualitative Research Topic Guide
Interviews with Professionals with a Relevant Role
e.g. with pregnant smokers, cessation services
V1.0: 05/07/2017

Interviews with professionals aim to explore issues around referral and recruitment to the trial, and perceived response among participants.

Interviews will be adapted depending on respondents' experience.
The topic guide is intended to ensure coverage of key topics, whilst at the same time giving respondents the freedom to express their own feelings and views as part of an open discussion.
****

INTRODUCTION
- Provide a reminder of the purpose and main focus of the trial
- Explain that focusing on opinions and experiences (not a test)
- Provide opportunity to ask questions
- Introduce the audio-recorder, underlining the importance of confidentiality
- Ask participant to complete consent form

A. BACKGROUND: OVERVIEW OF ROLE AND RELEVANT INVOLVEMENT IN TRIAL (also warm-up questions)
- General role
- Involvement with cessation in pregnancy
- Involvement in the trial

B. CONFIGURATION OF SERVICE AND CHARACTERISTICS OF PATIENT COMMUNITY
- Nature of service in which you work: how is it structured, where is it based, what staff groupings are involved
- Links between maternity service staff and smoking cessation staff or services: including knowledge, communication, referral process, feedback
- Characteristics of patient community served: level of affluence, degree to which urban/rural; age range, ethnicity, unemployment status, other?

C. TRIAL SET UP AND SUPPORT
- Explore knowledge and experience of changes made in anticipation of trial within own and related services
    - Communication and consultation regarding trial
    - Training
    - Working with other professionals
    - Facilities including access to consultation rooms, equipment, IT
    - Perception of response of colleagues in own as well as other services
    - Other challenges or opportunities not covered
    - Any perceived shortcomings in service preparations for trial

- Explore general experience of referral and recruitment of pregnant women to the trial then focus upon elements not already discussed. *Not all will be relevant to individual experience.*

<u>Initial booking visit (Midwives)</u>
- Asking pregnant women about their smoking
- Taking carbon monoxide readings (CO) readings and recording

- Asking permission to send information or dicussing automatic referral (where appropriate) to NHS stop smoking services
- Explore challenges and opportunities e.g. demanding appointment session, smokers' responses, preparation for this activity

First telephone contact with NHS Smokefree Pregnancy adviser (Smoking cessation staff)

- challenges of making contact by telephone (including out of hours)
- informing about the trial
- verbal permission to forward details to the Contact Centre
- perceived clients' response to service (e.g. thoughts on attending session, challenges to
- quitting)
- perceived clients' response to trial including reasons for participation (e.g. impact of incentives, random allocation)
- Process issues e.g. capacity issues in covering appointments
- Any prior awareness of using incentives for cessation and current trial

Consent call from NHS Stop Smoking Helpline (Contact Centre staff):

- Giving information about the trial
- Obtaining verbal consent
- Introducing and discussing random allocation to intervention or control groups
- Explore views of challenges experienced or anticipated e.g. contact successes, explaining complex issues, response to random allocation, perceived effect on engaging with services.

D. SUGGESTIONS FOR IMPROVING REFERRAL & RECRUITMENT

- Engaging women in the trial
  - Terms to describe the incentive scheme to pregnant women / what are key aspects women need to know/how best communicated
  - Any thoughts on what would make it easier/more attractive for women to engage in the trial/intervention
- What have been the barriers to referral and recruitment and what have been facilitators?
- Any ideas on what could be done to address barriers and enhance or extend facilitators?
- Any additional learning?

E. OVERVIEW

- Responses to concept of incentives - respondent's views and perceptions of others' views e.g. pregnant women, professionals and general public
  - Incentives as provided in current trial [provide overview of key stages within intervention and associated incentives]
  - Perceptions of any unintended outcomes
- Thoughts on incentives becoming a standard approach for supporting pregnant smokers to quit
- Any final thoughts on trial

For peer review only

CPIT 3 Process Evaluation

Nodes\\Revised Coding Framework

Coding Framework revised after testing by JM and IU August 2019

Name	Description
01, Job Role, professional background and experience	
a) Description of job role and relevant training	Including length of time in current post and professional background
b) Role within the trial	Including feelings about role within the trial
e) Professional standards or codes of conduct	
f) Importance of research evidence to role or practice	
02, Trial introduction and set-up	
a) How and when first heard about trial and from whom	
b) Perspectives of the trial team	

For peer review only - http://bmjopen.bmj.com/site/about/guidelines.xhtml

Name	Description
c) Initial thoughts and feelings about using incentives to encourage smoking cessation	Including interest in the trial
d) Initial thoughts and feelings about service or employer being involved with trial	
e) Decision-making about involvement in the trial	
f) Expectations of the trial	Including thoughts on execution of the research - also thoughts on CPIT phase 2 results
g) Preparations and training for CPIT	
03, Routine Service Characteristics	
a) Nature of booking appointment and midwifery care generally	Including impact of national policies on midwifery care.
b) Discussion of smoking within booking appointments	Where relevant to discussion of smoking also mention of CO monitoring
c) CO monitoring	
d) Criteria for and method of referral plus how women are first contacted by SSS	Also includes the typical number of referrals received each month by the service

For peer review only - http://bmjopen.bmj.com/site/about/guidelines.xhtml

Name	Description
e) Approach to and nature of support from smoking cessation service	Including level of engagement from women (prior to the trial). Also includes approach of midwives to support e.g specialist substance use midwives
f) Access to NRT and ability to prescribe directly to clients	
g) Monitoring of referral, engagement and outcomes	
h) Links between maternity staff and SSS	Including perspectives of the other group
i) Description and history of service or employing organisation	Also, description of management structures and organisational change prior to the trial. Description of how they feel about service including possible ownership. Also relationship with women - level of engagement
j) Discussion of local or national strategies with relevance to service or individual role	
04, Delivery and process of trial (feasibility)	
a) Introducing the trial to women referred	Including checking for eligibility
b) Consent call from ECHO	Including thoughts on randomisation to one of two groups

Name	Description
c) Face to face contact with adviser and setting a quit date	
d) Smoking status phone calls or research nurse visits for CO monitoring and triggering of incentives	
e) End of pregnancy smoking status, CO monitoring and cotinine testing	
f) Final smoking status check at 6 months post partum	
g) Client receiving or using financial incentives at key stages, practical issues	
h) Timing issues and any impact of delays	
05, Impact of the trial on services and women	
a) Maternity Service	
b) Smoking Cessation Service	
c) Women	

For peer review only - http://bmjopen.bmj.com/site/about/guidelines.xhtml

Name	Description
06, Making the trial work	
a) Information sharing and communication between trial staff and SSS	
b) Cooperation and compromise	
c) Support from the trial to the SSS	
07, Incentives and motivation	
a) Perspectives of acceptability of using incentives to promote smoking cessation in pregnancy	
i) Any awareness of incentives being used to change other behaviours	
ii) Feelings regarding the use of incentives	
iii) Type of incentive i.e financial versus other	
iv) Amounts and staged approach	

For peer review only - http://bmjopen.bmj.com/site/about/guidelines.xhtml

Name	Description
v) Thoughts on possible impact including length of time offered	
b) Perceived impact on client motivation to engage, to quit, to stay quit	
i) Overview of motivating factors for quit	
ii) Perceived impact of incentives	Including extent to which there has been greater engagement from women or the types of women who are accessing support
iii) Relative importance of incentives in context of other intervention factors	e.g. Role of smoking cessation adviser or CO monitoring
iv) Relative importance of incentives in context of other personal factors	e.g health, saving money, stress, smoking by partner or family and friends
v) Women's feedback on incentives	Including what incentives have been used on
vi) Any impact on professional appreciation of using incentives	i.e as a selling point

For peer review only - http://bmjopen.bmj.com/site/about/guidelines.xhtml

Name	Description
08, Local context and characteristics of population served by smoking cessation service	
a) Geography in terms of rural or urban setting	
b) Socio-economic background	
c) Age	
d) Ethnic mix	
f) Other important characteristics of local population	
09, Recommendations for improving the trial and learning for future rollout	
a) Recommendations to improve recruitment to the trial	
b) Recommendations to change type, amount or timing of incentives	
c) Recommendations to improve follow up of participants including monitoring	

For peer review only - http://bmjopen.bmj.com/site/about/guidelines.xhtml

Name	Description
d) Learning useful for future rollout from perspective of participants	
e) Learning useful for future rollout from perspective of smoking cessation service	
f) Views or suggestions for the process evaluation	
g) Learning points for running a trial within a service or organisation	
h) What worked in Glasgow and comparison with current sites	
10, Impression of Interview	Manner and tone of participant
11 Miscellaneous	
12 Barriers to Quitting	
13, Experience of supporting pregnant women to stop smoking*	
14, Progress of trial*	
14.1 Changes to the trial process	

For peer review only - http://bmjopen.bmj.com/site/about/guidelines.xhtml

Name	Description
15, Working with (Name of call centre company managing trial consent process)*	

* New nodes added by JM after revised framework was finalised in August 2019

For peer review only

For peer review only - http://bmjopen.bmj.com/site/about/guidelines.xhtml

COREQ Checklist: ***Usual care in a multi-centre randomised controlled trial of financial incentives for smoking cessation in pregnancy: qualitative findings from a mixed methods process evaluation***

No	Item	Presence (Y/N)	Page Number
Domain 1: Research Team and reflexivity			
Personal Characteristics			
1	Interviewer/facilitator	Y	6
2	Credentials	Y	15
3	Occupation	Y	15
4	Gender	Y	6
5	Experience and training	Y	6/15
Relationships with participants			
6	Relationship established	Y	6
7	Participant knowledge of the interviewer	Y	6
8	Interviewer characteristics	N	N/A
Domain 2: Study Design			
Theoretical framework			
9	Methodological orientation and theory	Y	4 and 6
Participant selection			
10	Sampling	Y	6
11	Method of approach	N	N/A
12	Sample size	Y	4/5
13	Participation	N	N/A
Setting			
14	Setting of data collection	Y	6
15	Presence of non-participants	Y	6
16	Description of sample	N	N/A
Data Collection			
17	Interview guide	Y	4 (supplementary files)
18	Repeat interviews	N	N/A
19	Audio-visual recording	N	N/A
20	Field notes	N	N/A
21	Duration	Y	6
22	Data saturation	N	N/A
23	Transcripts returned	N	N/A
Domain 3: Analysis and Findings			
Data Analysis			
24	Number of data coders	Y	6
25	Description of coding tree	Y	6 (supplementary file)
26	Derivation of themes	Y	6
27	Software	Y	6
28	Participant checking	N	N/A
Reporting			
29	Quotations presented	Y	8-12
30	Data and findings consistent	Y	7-12
31	Clarity of major themes	Y	7, 8
32	Clarity of minor themes	Y	8-12

For peer review only
